# Novel tools for genomic modification and heterologous gene expression in the phylum *Planctomycetota*

**DOI:** 10.1007/s00253-025-13462-w

**Published:** 2025-03-31

**Authors:** Tom Haufschild, Jonathan Hammer, Nico Rabold, Veronika Plut, Christian Jogler, Nicolai Kallscheuer

**Affiliations:** 1 Department of Microbial Interactions, Institute for Microbiology, Friedrich Schiller University, 07743 Jena, Germany; 2 Cluster of Excellence Balance of the Microverse, Friedrich Schiller University, 07743 Jena, Germany

**Keywords:** Planctomycetes, *Planctopirus limnophila*, Fluorescent proteins, Inducible gene expression, Gene inactivation, Cell biology

## Abstract

**Abstract:**

Members of the phylum *Planctomycetota* possess a plethora of intriguing and hitherto underexplored features including an enlarged periplasmic space, asymmetric cell division (“budding”), and a mostly undiscovered small molecule portfolio. Due to the large phylogenetic distance to frequently used and easily genetically accessible model bacteria, most of the established genetic tools are not readily applicable for the here-investigated bacterial phylum. However, techniques for targeted gene inactivation and the introduction of heterologous genes are crucial to investigate the cell biology in the phylum in greater detail. In this study, the targeted genomic modification of model planctomycetes was achieved by enforcing two types of homologous recombination events: simultaneous double homologous recombination for the deletion of coding regions and insertion-duplication mutagenesis for the introduction of foreign DNA into the chromosome. Upon testing the expression of commonly used fluorescent protein-encoding genes, many of the tested native promoters could not be harnessed for variation of the expression strength. Since also four commonly used inducible gene expression systems did not work in the tested model strain *Planctopirus limnophila*, a native rhamnose-dependent transcriptional regulator/promoter pair was established as an inducible expression system. The expanded molecular toolbox will allow the future characterization of genome-encoded features in the understudied phylum.

**Key points:**

• *Two recombination methods were used for the genetic modification of planctomycetes*

• *Commonly used fluorescent proteins are functional in model planctomycetes*

• *A rhamnose-dependent regulator was turned into an inducible expression system*

**Supplementary Information:**

The online version contains supplementary material available at 10.1007/s00253-025-13462-w.

## Introduction

Bacteria of the phylum *Planctomycetota* are increasingly recognized for their intriguing cell biology and potential biotechnological application. Despite being the fourth most prevalent bacterial phylum in soil (Delgado-Baquerizo et al. 2018), previous research has primarily focused on *Planctomycetota* in aquatic environments. In such ecosystems, members of the phylum commonly associate with various marine phototrophs (Bengtsson et al. 2012; Bondoso et al. 2014; Lage and Bondoso 2014; Vollmers et al. 2017), where they form dominant biofilms (Bengtsson and Øvreås 2010; Kohn et al. 2020) and metabolize polymeric carbon substrates (Jeske et al. 2013; Lachnit et al. 2013). Their prevalence on nutrient-rich surfaces in otherwise oligotrophic ecosystems, as for example oceans, is intriguing given their relatively slow growth compared to microbial competitors, e.g., members of the phylum *Pseudomonadota* (Frank et al. 2014; Wiegand et al. 2018).

In a joint effort, our team and others have isolated and described more than 100 novel strains from diverse environments in the last decade that have been added to the current open collection of ca. 130 strains (Devos et al. 2020; Kallscheuer et al. 2024; Wiegand et al. 2020). Previous investigations of their cell morphology suggested exceptions to existing models of diderm bacteria (Boedeker et al. 2017; Jeske et al. 2015). For example, they possess distinctive crateriform structures potentially involved in polysaccharide uptake (Boedeker et al. 2017). An enlarged periplasmic space might facilitate the subsequent digestion of internalized polysaccharides. Asymmetric cell division independent of the hallmark protein FtsZ is another distinctive feature of most members of the phylum (Jogler et al. 2012; Rivas-Marin et al. 2020; Wiegand et al. 2020). While planctomycetal cell walls contain peptidoglycan (Jeske et al. 2015; van Teeseling et al. 2015), genes involved in its biosynthesis are only partially present in planctomycetal genomes (Mahajan et al. 2020; Wiegand et al. 2020). In the limnic species *Planctopirus limnophila*, the peptidoglycan biosynthesis genes have even been shown to be non-essential (Rivas-Marin et al. 2023).

Bioprospection studies published in the last 5 years have started to uncover the untapped small molecule portfolio of the phylum (Graça et al. 2016; Jeske et al. 2016), which includes molecules with potential health-promoting bioactivities (Belova et al. 2020; Calisto et al. 2019; Kallscheuer and Jogler 2021). The ability to produce bioactive compounds, their large genomes, and the high number of genes of unknown function (Kallscheuer and Jogler 2021) make phylum members promising candidates for drug discovery and development. Recent discoveries include stieleriacines, potential biosurfactants (Kallscheuer et al. 2020; Sandargo et al. 2020), carotenoid pigments (Kallscheuer et al. 2019; Santana-Molina et al. 2022), an aromatic plant toxin (Panter et al. 2019), and alkylresorcinols of yet unknown function (Milke et al. 2024).

These recent discoveries in cell biology and small molecule biochemistry mark a transition phase from the descriptive characterization of novel strains to understanding fundamental molecular machineries and exploring potential biotechnological applications. The predicted ability of characterized strains to degrade complex polysaccharides suggests additional applications in industrial processes, such as biomass conversion and bioremediation (Boedeker et al. 2017; Klimek et al. 2024).

So far, genetic tool development for *Planctomycetota* has lagged behind other bacterial phyla. Main obstacles were the limited availability of axenic cultures, the requirement of complex media compositions, the natural low growth rate (typical generation times between 8 and 100 h), the aggregation behavior, and the natural resistance to common antibiotics (Godinho et al. 2019; Ivanova et al. 2021; Kallscheuer et al. 2021). Genetic tools are available for four species belonging to three different orders within the phylum *Planctomycetota* (Fig. S1) (Jogler et al. 2011; Rivas-Marín et al. 2016). Genetic manipulation was mainly based on random transposon-mediated insertion mutagenesis (Rivas-Marin et al. 2023) and the introduction of reporter genes (Rivas-Marín et al. 2016; Boedeker et al. 2017) as well as modification at single genomic loci (Erbilgin et al. 2014; Wiegand et al. 2020). To accelerate the research progress in this phylum, more robust genetic tools allowing the target-specific genetic manipulation of planctomycetes are urgently needed, and therefore must be established and further optimized for these bacteria.

**Fig. 1:**
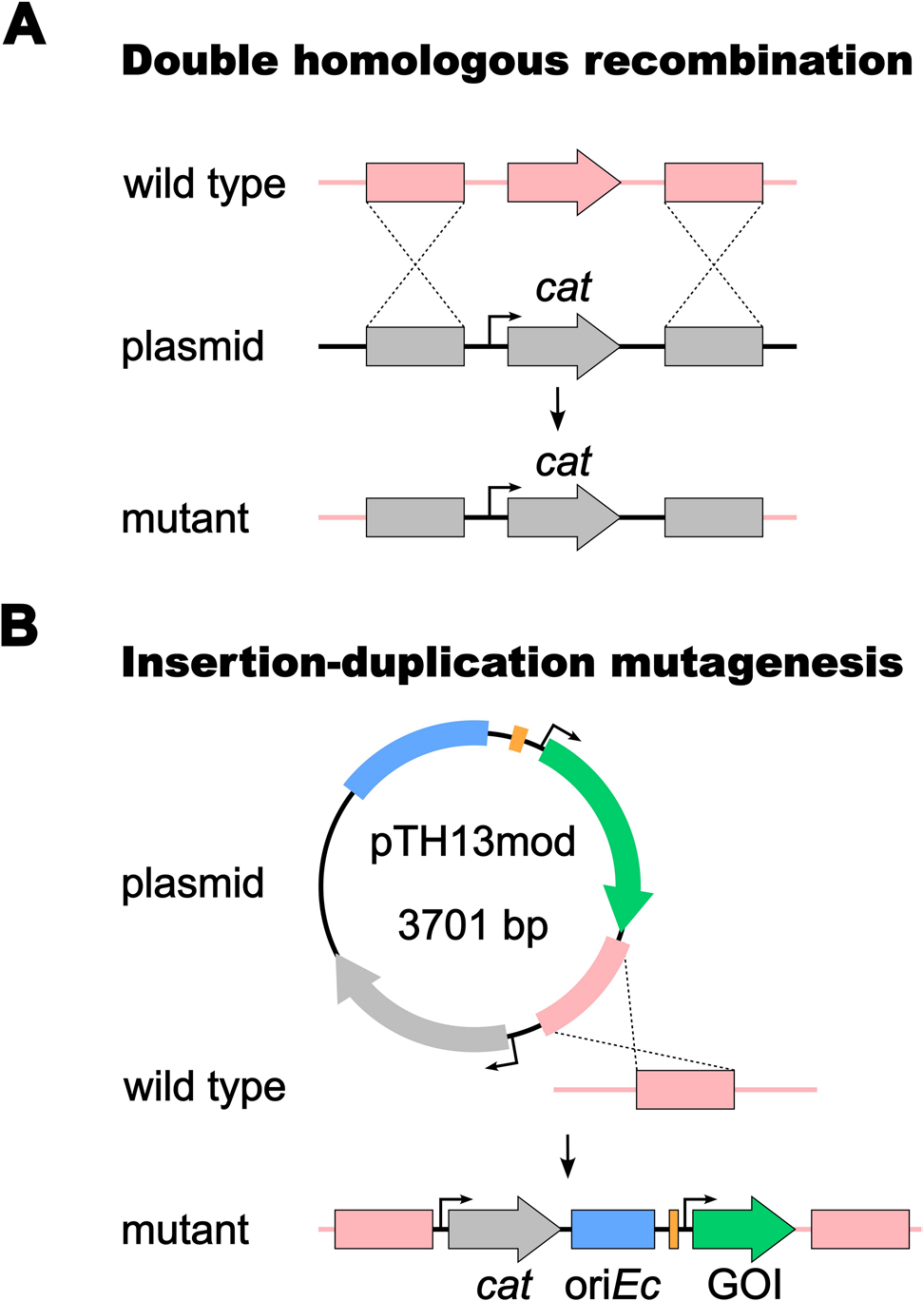
Homologous recombination strategies used for gene inactivation and introduction of foreign genes. **A** Simultaneous double homologous recombination based on two recombination events between an up- and downstream region of a genomic target region (grey boxes). **B** Insertion-duplication mutagenesis based on a single homology region used to insert entire plasmid cassettes into the chromosome. The *cat* gene (encoding chloramphenicol acetyltransferase) as resistance marker is shown exemplarily. GOI, gene of interest; ori, origin of replication

In this study, an improved molecular toolbox is presented that is based on two homologous recombination strategies (Fig. 1) applicable for gene inactivation and introduction of foreign genes at defined loci. The combination of parts of a native regulatory circuit, fluorescent reporter genes, and selection markers was exploited for the construction of genetically modified strains of the limnic species *P. limnophila* and the marine species *Stieleria maiorica*.

## Materials and methods

### Medium preparation and cultivation

*Escherichia coli* DH5α was used for plasmid constructions and was cultivated in LB medium (5 g/L yeast extract, 10 g/L tryptone, 5 g/L NaCl) at 37 °C. The limnic model planctomycete *P. limnophila* DSM 3776^ T^ was cultivated in limnic M3 medium (synonym: M3H NAG AFW), while the marine strain *S. maiorica* Mal15^T^ was cultivated in marine M3 medium (synonym: M3H NAG ASW) (Wiegand et al. 2020). If required, kanamycin (50 mg/L), chloramphenicol (34 mg/L), or spectinomycin (100 mg/L) were added to the media. Solidified media were prepared by the addition of 15 g/L agar. For the planctomycete media, the agar was washed three times with ddH_2_O, autoclaved separately (121 °C, 20 min), and added after cooling down to < 60 °C. Bacterial growth was followed by measuring the optical density at 600 nm (OD_600_). For cultivation experiments with wild-type and genetically engineered strains of *P. limnophila* and *S. maiorica*, strains were first streaked on limnic and marine M3 agar plates, respectively, with the appropriate antibiotic (when applicable). Material from the plates was used for the inoculation of a preculture. All main cultures were inoculated from precultures to the indicated OD_600_.

### DNA amplification and construction of plasmids

All bacterial strains and plasmids used in this study as well as their relevant characteristics are listed in Table S1. Oligonucleotides used in this study are listed in Table S2. Standard protocols of molecular cloning, such as PCR, DNA restriction, and ligation (Sambrook and Russell 2001), were carried out for recombinant DNA work. The amplification of DNA fragments for subsequent cloning into plasmids was performed using the Q5 polymerase Master Mix (New England Biolabs) based on the manufacturer’s protocol. Oligonucleotides were obtained from Metabion. The PCR clean-up was conducted using the NucleoSpin Gel and PCR Clean-up Mini Kit (Macherey–Nagel). Colony material of *P. limnophila* or *S. maiorica* or plasmids harboring the desired genes were used as template. FastDigest enzymes used for restriction and alkaline phosphatase (FastAP) for vector dephosphorylation were obtained from Thermo Scientific. The Rapid DNA Ligation Kit (Thermo Scientific) was used for ligation reactions and the constructed plasmids were isolated using the GeneJET Plasmid Mini-Prep kit (Thermo Scientific). Check-PCR was performed with the DreamTaq Green PCR Master Mix (Thermo Scientific) with the following program: initial denaturation 5 min, 95 °C; 33 cycles of denaturation (95 °C, 20 s), annealing (58 °C, 15 s) and elongation (72 °C, 1 min/kb), and a final elongation step (95 °C, 5 min). All constructed plasmids were verified by check-PCR using primers that bind outside of the inserted sequences and by DNA sequencing at Macrogen Europe.

### Preparation of electrocompetent cells and transformation

Electrocompetent cells of *P. limnophila* were prepared from 50 mL of an exponentially growing culture (OD_600_ of 0.5–0.7) in limnic M3 medium. For *S. maiorica* Mal15^T^, 200 mL of an exponentially growing culture (OD_600_ of 0.5–0.7) in marine M3 medium was harvested. Cells were harvested by centrifugation (4600 × *g*, 4 °C, 20 min) and washed twice with sterile 10% (v/v) glycerol. Cells obtained after the second washing step were mixed with (linearized) plasmid DNA and transferred to a Gene Pulser Electroporation Cuvette with 0.2 cm gap (Biorad). The electroporation was performed with a GenePulser Xcell (Biorad) with the following parameters: 2500 V, 25 µF, 200 Ω, pulsing time 5 ms. The cell suspension was immediately transferred to 4 mL limnic M3 medium (*P. limnophila*) or marine M3 medium (*S. maiorica*) and cultivated for 3 h at 28 °C on a rotary shaker. The cells were harvested by centrifugation (4600 × *g*, 20 °C, 6 min) and ultimately streaked on plates containing the appropriate antibiotics that were subsequently incubated at 28 °C. Colonies obtained after 7–10 days were re-streaked on antibiotic-containing agar plates and checked for the presence of the expected modification in the genome using check-PCR.

### Gene inactivation by two simultaneous crossing-over events

For the targeted gene inactivation by two simultaneous crossing-over events, the flanking regions up- and downstream of the coding sequence of a specific gene (approximately 1500 bp each) were amplified and subsequently cloned into the plasmid pCJ003x(kan), pCJ003x(cat), or pCJ003x(spec), depending on the chosen selection marker. The amplified homology regions still included the first and last 24 nucleotides of the coding sequence of the inactivation target. In the final plasmid, the inserted homology arms flank the resistance gene including its promoter. Prior to the transformation of electrocompetent cells, 2 µg of plasmid DNA was linearized with a single-cutting restriction enzyme that cleaves outside of the assembled insert region (upstream homology region–resistance gene–downstream homology region) and was purified with the NucleoSpin Gel and PCR Clean‑up Kit (Macherey–Nagel).

### Introduction of heterologous genes by insertion-duplication mutagenesis

For testing the expression of heterologous genes, entire plasmid cassettes were inserted into the genome. This was achieved using an insertion-duplication strategy based on a single crossing-over event. For this purpose, a ca. 1300-bp fragment amplified from the genome of *P. limnophila* was inserted into the plasmid pASK-IBA3C (IBA Lifesciences) that was previously modified by removal of the anhydrotetracycline-inducible *tetA* promoter. The resulting plasmid pTH13mod was used in subsequent cloning steps for the insertion of constitutive and inducible promoters in combination with fluorescent reporter genes. Preparation of competent cells and transformation of *P. limnophila* were performed in the same manner as described above, but 2 µg of circular plasmid DNA was used for the transformation.

### Isolation of genomic DNA, Illumina sequencing, and read mapping

DNA extraction and quality control were performed according to a previously published workflow (Wurzbacher et al. 2024). Illumina NovaSeq sequencing of *P. limnophila* DSM 3776^ T^ wild type and mutant strains was performed by Eurofins Genomics (Luxemburg). The obtained reads (per strain approx. 5 million read pairs, 2 × 150 bp) were uploaded to the Galaxy Europe web server (The Galaxy Community 2022). All reads passed the quality check with FastQC version 0.11.9 (https://github.com/s-andrews/FastQC) and were further processed without additional trimming and filtering.

The reads of *P. limnophila* DSM 3776^ T^ were used to polish the NCBI reference genome (accession number: ASM9210v1). Read mapping to the reference was done using BWA-MEM2 version 2.2.1 (Li and Durbin 2009, 2010) with the “Simple Illumina Mode”. Genome polishing was performed using Pilon version 1.20 (Walker et al. 2014). The polished reference genome of the *P. limnophila* was annotated with prokka version 1.14.6 (Seemann 2014). To analyze the gene inactivation mutants, the sequencing reads of the mutant strains were mapped to the polished reference genome of *P. limnophila* using BWA-MEM2 version 2.2.1 (Li and Durbin 2009, 2010) with the “Simple Illumina Mode”. The results were visualized with the IGV genome browser (Robinson et al. 2017) and manually analyzed.

### Fluorescence microscopy setup and image analysis

Cell mounting, microscopy, post-processing, and image analysis were performed as described previously (Haufschild et al. 2024) with minor modifications. Briefly, 2 µL of the cultures were placed on 1% (w/v) agarose cushions on object slides and covered with a coverslip. Cells were imaged with a Ti2 inverse microscope (Nikon) equipped with a Nikon N Plan Apo λ 100x/1.45 Oil objective, a Hamamatsu Orca-flash 4.0 LT Plus camera, and a Lumencor light source. Images were split in FIJI (Schindelin et al. 2012) and analyzed in BacStalk (Hartmann et al. 2020) with 0.065 μm as pixel size, 25 pixels as cell size, and 15 pixels as minimal cell size for cell segmentation and mean fluorescence intensities per cell were extracted. The obtained data was visualized with SuperPlotsOfData (Goedhart 2021).

### Fluorescence microscopy of fluorescent reporters

For microscopy of cells of *P. limnophila* and *S. maiorica* harboring a chromosomally integrated fluorescent reporter, the following combination of fluorescent reporters, LED wavelengths, and filter cubes were used: msfTurquoise2ox (440 nm, Semrock CFP-2432C); mNeonGreen, GFPmut2, and msfGFP (470 nm, Semrock FITC-3540C), mVenus (508 nm, Semrock YFP-2427B), mCherry (555 nm, mCherry-C), and HaloTag with OregonGreen Ligand (470 nm, Semrock FITC-3540C). For staining and washing of *P. limnophila* cells expressing the HaloTag-encoding gene, the manufacturer’s recommendations were followed. Images were transferred to FIJI where intensity and brightness were adjusted and scale bars were added.

### Analysis of gene expression strength of native promoters from *P. limnophila*

Cells of *P. limnophila* strains constructed for the gene expression analysis were cultivated for 2 days in precultures and main cultures were inoculated to an initial OD_600_ of 0.1. After 1 day of incubation, the OD_600_ was measured; 500 µL of cells was centrifuged for 3 min at 17,000* g*, washed with sterile distilled water; and fluorescence intensity of 150 µL culture was measured with an Infinite MPlex 200 plate reader (Tecan). Prior to measurement, the culture was homogenized by 5-s orbital shaking followed by 8-s linear shaking. Measurement settings were as follows: 499-nm excitation wavelength (9-nm bandwidth), 530-nm emission wavelength (20-nm bandwidth), gain value of 14, 15 flashes, and 20-µs integration time. Obtained fluorescence signals were corrected for the autofluorescence of the medium and normalized to the OD_600_ before mean values were calculated. Negative values for fluorescence intensities obtained after autofluorescence subtraction were regarded as a fluorescence intensity of zero. The data was further normalized to the fluorescence intensity of the *P*_*gap*_ construct (= 100%). The fluorescence intensity values measured with the MPlex platereader were visualized with RStudio (RStudio 2022) employing the ggplot2 package (Wickham 2016). For fluorescence microscopy, LED and filter settings were used as described above for mNeonGreen, the exposure time was set to 400 ms for all samples. The mean fluorescence intensity of cells (in total 300 cells per condition) was analyzed as described above.

### Analysis of the rhamnose-inducible gene expression system in *P. limnophila*

*P. limnophila* strains with either the empty vector control or the plasmid bearing the rhamnose-inducible construct *P*_*pvmA*_*-pvmA-P*_*pvmB*_*-msfgfp* introduced into the chromosome were inoculated in medium containing glucose and *N*-acetyl glucosamine (NAG). After 2 days of growth, cells were incubated to an OD_600_ of 0.05 in limnic M3 medium with glucose, but without NAG. Two days later, cells were inoculated to an OD_600_ of 0.1 (main culture) in limnic M3 medium containing the respective conditions comprising either 0.2% (w/v) rhamnose, 0.1% (w/v) rhamnose and 0.1% (w/v) glucose, 0.2% (w/v) glucose, or none of the two sugars. After 24 h, cells were imaged with the setup described above using the LED/filter cube setting for msfGFP; exposure time was the same for all samples. For image processing, fluorescence brightness and contrast settings of all images were adjusted manually to the same values and scale bars were added using FIJI. Image analysis was conducted as described above analyzing 400 cells per condition in total (200 cells of two biological replicates). The same experimental and analysis design was used for the rhamnose concentration-dependence experiment. The only difference were the sugar concentrations (w/v) added to the medium, either 0.2% glucose, 0.05%, 0.1%, 0.2%, 0.5%, or 1% rhamnose. In total, four replicates with 150 cells per replicate were analyzed.

## Results

### Construction of single gene deletion mutants in limnic and marine model planctomycetes

**Fig. 2:**
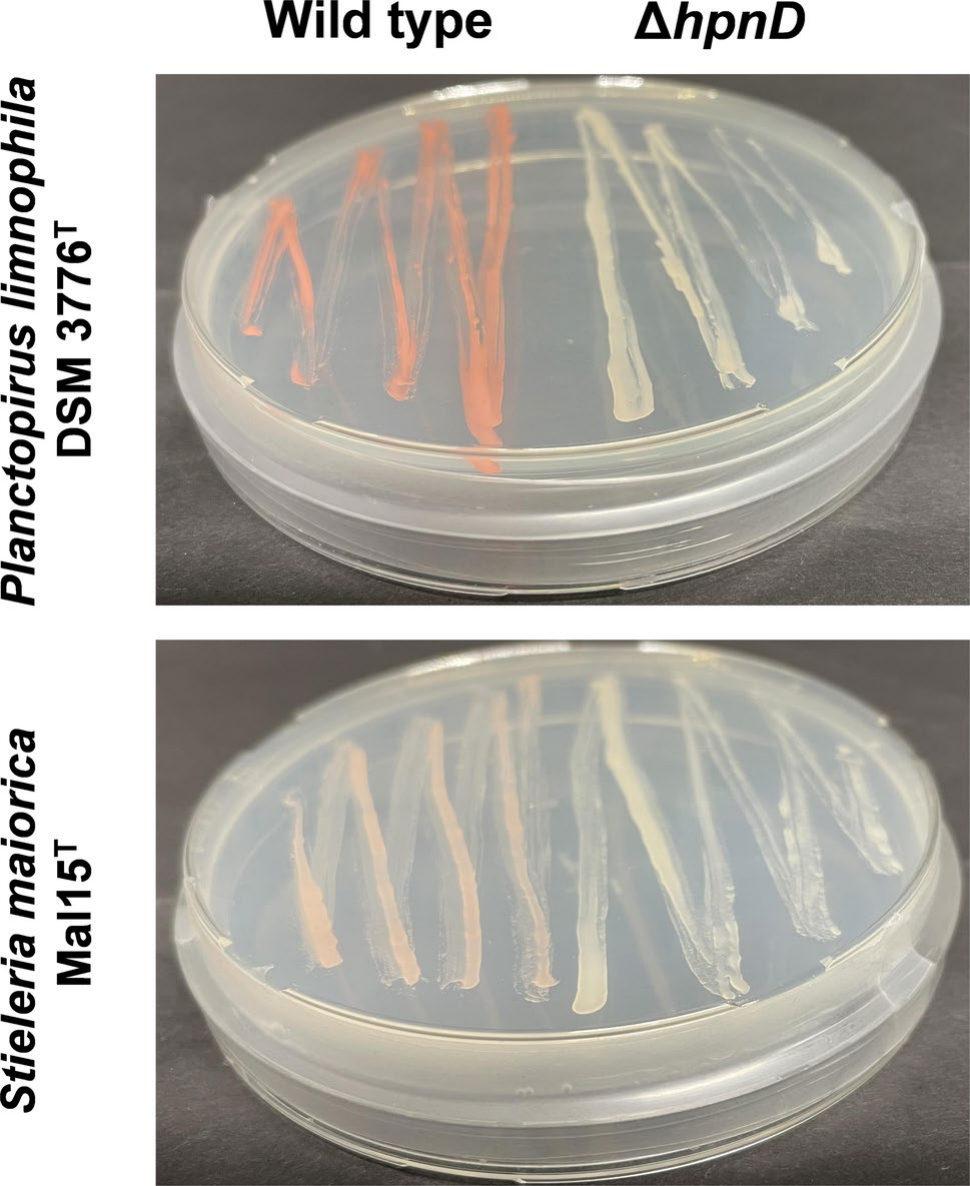
Target-specific inactivation of genes. The presqualene diphosphate synthase HpnD catalyzes the initial step during C_30_ carotenoid biosynthesis in planctomycetes. The deletion of the encoding gene abolished carotenoid formation and yielded white colonies in *P. limnophila* and *S. maiorica*. The wild type of both species is shown for comparison

The easiest way to investigate the role of non-essential protein-coding genes is the construction of respective gene inactivation mutants by interrupting the open reading frame or removing it (Fig. S2). Depending on the inactivation target, the deletion can result in a detectable phenotype, e.g., the inability to degrade a certain carbon source or to form a characteristic pigment. The presqualene diphosphate synthase HpnD catalyzes the initial step during synthesis of the triterpene squalene from farnesyl pyrophosphate. Squalene serves as precursor for C_30_ carotenoid biosynthesis in *P. limnophila* (Santana-Molina et al. 2022). Hence, deletion of the encoding gene should abolish carotenoid formation and yield white colonies. The deletion was tested in the type strains of the pink-pigmented and limnic model species *P. limnophila* (*hpnD* locus tag Plim_2244, UniProt entry D5SNF6) and the salmon-colored marine species *S. maiorica* (*hpnD* locus tag Mal15_66750, UniProt entry A0A5B9MNP2). A kanamycin resistance gene was used for the selection of recombinant clones of *P. limnophila* while inactivation in *S. maiorica* required a chloramphenicol resistance gene (*cat*) due to resistance of the species to aminoglycosides. One week after transformation of the two strains by electroporation, approximately 100 and 20 recombinant clones were obtained for *P. limnophila* and *S. maiorica*, respectively. All obtained colonies lacked the pink or salmon pigmentation of the respective wild-type strains indicating that the recombination event occurred at the correct genomic location in all obtained colonies. This was also confirmed by check PCR performed with three colonies per strain that were re-streaked to check for maintenance of the resistance marker. The linear DNA carrying the flanking homology regions of the inactivation targets successfully enforced simultaneous homologous recombination events (Fig. 1A) that ultimately led to the exchange of *hpnD* with the resistance marker gene (Fig. 2, S3). The recombination did not require any foreign recombinases.

### Modifications at up to three genomic loci can be combined in *P. limnophila*

The here-used gene inactivation strategy does not include a counterselection step to remove the resistance marker gene. Since such a marker is currently not available for the phylum, the resistance marker gene needs to remain in the genome of the recombinant strain. However, in case that a strain is susceptible to other commonly used antibiotics with established resistance markers, it should be possible to combine modifications at different loci in one strain. Unfortunately, *S. maiorica* turned out to be naturally resistant to kanamycin, spectinomycin, tetracycline, and β-lactam antibiotics (such as ampicillin and carbenicillin) and was not considered for testing the introduction of combined genetic modifications at two or more loci. In contrast, the limnic *P. limnophila* is susceptible to kanamycin, spectinomycin, and chloramphenicol and only resistant to β-lactam antibiotics.

After an inspection of the *P. limnophila* genome for inactivation targets that have similar putative functions but are not essential under laboratory-scale cultivation conditions, genes encoding PilQ-like proteins turned out to be suitable candidates. PilQ proteins are involved in the formation of type IV pili (T4P) by assembly into homo-multimeric structures constituting the outer membrane pore of the T4P apparatus (Berry et al. 2012). From constructed single gene deletions, it was clear that the three putative *pilQ* genes Plim_0794, Plim_3284, and Plim_3688 are not essential under standard cultivation conditions and that the tested resistance markers for chloramphenicol and spectinomycin are functional in *P. limnophila*. The expected sequence of these strains was first confirmed by check-PCRs and later via genome sequencing and read mapping to the modified loci. Off-target effects could thereby be excluded as well (Fig. S4A). By the subsequent deletion of the three genes, the triple deletion mutant *P. limnophila* ΔPlim_3688::*kanR* ΔPlim_0794::*cat* ΔPlim_3281::*specR* could be obtained. The expected genotype of the strain was confirmed by check PCR-based amplification of the modified loci (Fig. S4B). After having confirmed that deletion mutants can be obtained in the way described, the cloning plasmids pCJ003x(kan), pCJ003x(cat), and pCJ003x(spec) that were used for the insertion of the homology regions flanking the inactivation target were modified. Plasmid sequences not required for replication in *E. coli* were removed and an additional terminator sequence downstream of the resistance gene was introduced along with improved multiple cloning sites for the insertion of the upstream and downstream homology regions. The stream-lined plasmids pDEL(kan), pDEL(cat), and pDEL(spec) (Fig. S5) will be used for the future construction of deletion mutants in planctomycetes.

### Commonly used fluorescent reporters are functional in planctomycetes

**Fig. 3:**
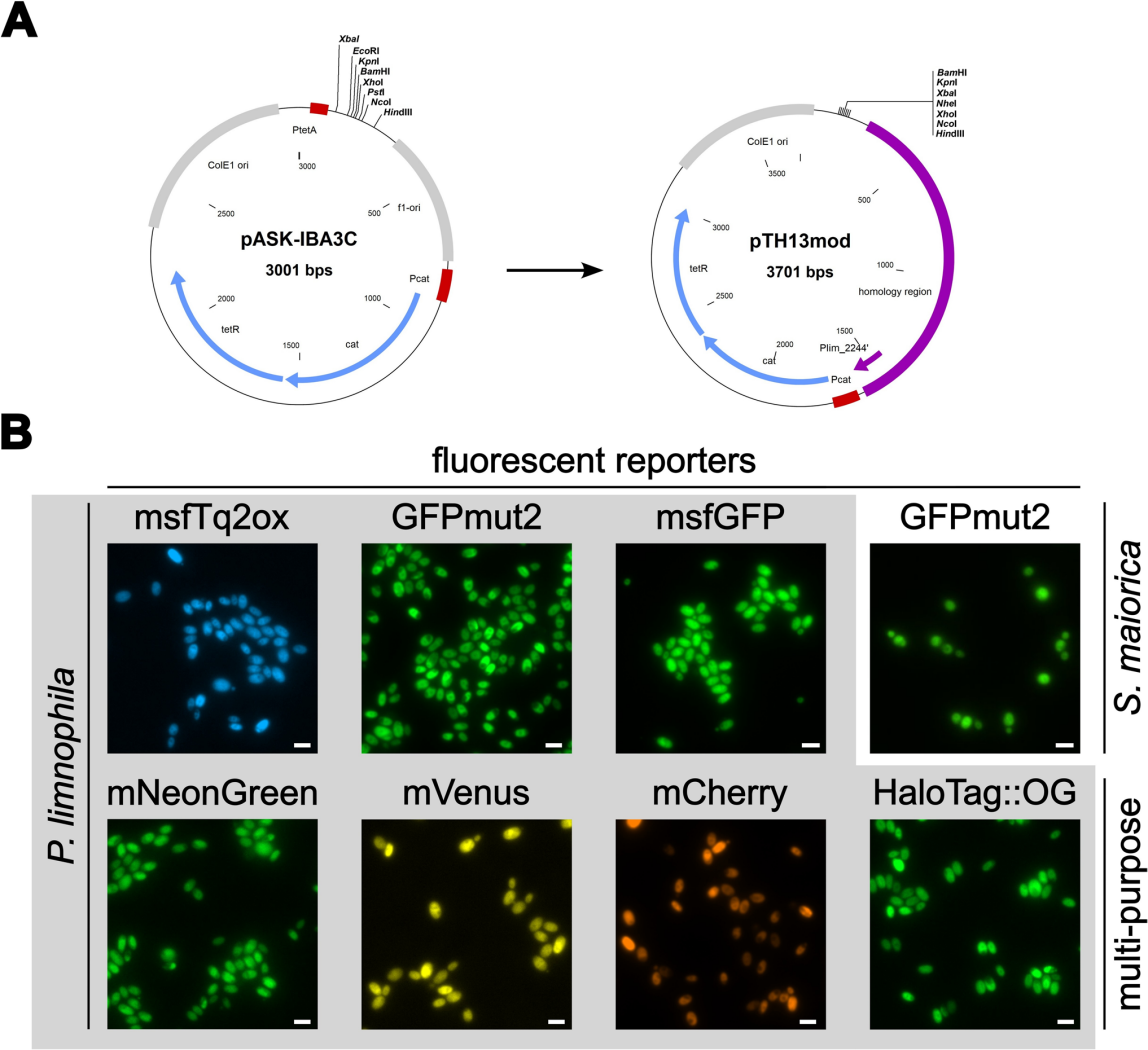
Duplication-insertion mutagenesis in *P. limnophila* results in the expression of various fluorescent protein-encoding genes. **A** The expression vector pASK-IBA3C was used for the construction of pTH13mod by removal of *P*_*tetA*_ and the f1-ori and replacement for an improved multiple cloning site and a region with homology to the *hpnD* locus of *P. limnophila* (purple). Unique restriction sites used for cloning are shown in the plasmid maps. **B** Epi-fluorescence images of *P. limnophila* cells show the expression of various reporter genes. *S. maiorica* with a stably integrated *gfpmut2* gene was constructed with a modified protocol of the gene deletion strategy via two homologous recombination events (see text for details). OG, Oregon Green

In previous studies, green fluorescent protein (GFP)-encoding genes have been introduced into four planctomycetotal species (Boedeker et al. 2017; Jogler et al 2011, Rivas-Marín et al. 2016). Although this led to a constitutive expression of the GFP-encoding gene as a weak dimer in the cytoplasm, the strategy is not well-suited for detailed protein function, localization, or interaction studies. Instead, studies employing wide-field and super-resolution microscopy methods based on monomeric fluorescent proteins are essential to address biological questions and require targeted modifications in the genome. For this reason, an insertion-duplication mutagenesis strategy was applied for the introduction of entire plasmids into the genome of *P. limnophila* using site-specific single homologous recombination (Fig. 1B). The starting point for these experiments was the empty vector pASK-IBA3C, a plasmid originally constructed for an inducible gene expression in *E. coli*. Since the *hpnD* gene locus Plim_2244 is accessible for genetic modifications, a 1300-bp sequence covering the upstream flanking region and the first 150 bp of the *hpnD* open reading frame was cloned into the plasmid outside of its multiple cloning site. The inserted DNA from *P. limnophila* functions as a “landing site” for the introduction of the entire plasmid into the genome. The anhydrotetracycline-inducible promoter from the original plasmid was removed to yield pTH13mod (Fig. 3A). To test the insertion into the chromosome of *P. limnophila*, the native constitutive promoter of the glyceraldehyde-3-phosphate dehydrogenase gene (*P*_*gap*_) and the previously tested *gfpmut2* were cloned into the plasmid yielding pTH13mod_Pgap-gfpmut2. The chosen promoter-reporter combination was already shown to be functional in a previous study (Boedeker et al. 2017). After transformation of electrocompetent *P. limnophila* cells, a low number of colonies (< 10) per plate was obtained. Microscopic analyses confirmed the expression of the gene *gfpmut2* in the three tested clones of the recombinant strain. The fluorescence signal occurred in the cytoplasm whereas invaginations of the periplasm were not stained by the cytoplasmic GFPmut2 (Fig. 3B). To expand the set of functional fluorescent proteins, a range of different commonly used proteins was tested (Table 1). Candidates were chosen based on various criteria including brightness, multimerization, activity in the cytoplasm and/or periplasm, and suitability as partner for translational fusions or for the use with exchangeable ligands (enabling pulse-chase labelling or the use of high-resolution fluorescence microscopy ligands). The selected set included genes encoding msfTurquoise2ox (msfTq2ox), mNeonGreen, mVenus, mCherry, msfGFP, and the multi-purpose HaloTag that were individually inserted into the genome of *P. limnophila*. Like GFPmut2, all other tested reporter proteins folded and matured properly as confirmed by fluorescence signals in the cytoplasm (Fig. 3B).

**Table 1:** Information on used fluorescent proteins

Fluorescent protein	Excitation/emission wavelength (nm)	Oligomerization	Activity in the periplasm	References
GFPmut2	481/507	Weak dimer	No	Cormack et al. 1996 Zacharias et al. 2002
msfGFP	488/510	Monomer	Yes	Pédelacq et al. 2006 Zacharias et al. 2002 Dinh and Bernhardt 2011
msfTq2ox	434/474 (taken from mTq2)	Monomer	Yes	Goedhart et al. 2012 Zacharias et al. 2002 Meiresonne et al. 2019
mNeonGreen	506/517	Monomer	Yes	Shaner et al. 2013 Zacharias et al. 2002 Meiresonne et al. 2017
mCherry	587/610	Monomer	Yes	Shaner et al. 2004 Zacharias et al. 2002 Meiresonne et al. 2019
mVenus	515/527	Monomer	No	Kremers et al. 2006 Zacharias et al. 2002

### Construction of a *Stieleria maiorica* strain with constitutive expression of gfpmut2

The handling of *S. maiorica* in the lab is quite challenging. The type strain Mal15^T^ forms large amounts of extracellular polymeric substance (EPS) which renders it difficult to harvest cells by centrifugation. However, a minimum of two washing steps is crucial to remove salts from the marine medium prior to the electroporation. From 200 mL culture broth, only a tiny cell pellet remained after the two washing steps and most cells embedded in the EPS were lost during removal of the supernatant. Although the pTH13mod-based insertion-duplication mutagenesis may in principle also work for *S. maiorica*, the protocol was not followed for this strain. This is due to the following reasons: (i) The insertion of entire plasmid cassettes in *P. limnophila* worked at least one order of magnitude worse than the enforced double homologous recombination strategy and it was not possible to compensate this by the collection of sufficient cells of *S. maiorica* after centrifugation; (ii) plasmids introduced by insertion-duplication mutagenesis can spontaneously excise from the genome by reversal of the homologous recombination event and this can only be prevented by maintaining the selection pressure (supplementation of antibiotics). Since the *gfpmut2*-expressing strain is planned to be used for co-cultivation experiments with antibiotic-susceptible interaction partners, a modified version of the double homologous gene deletion strategy was used for strain construction. For this purpose, the *gfpmut2* gene was used and the *hpnD* locus was targeted. The final plasmid used for *S. maiorica* strain construction contained the following insert: upstream homology arm of *hpnD* (ca. 1500 bp)–promoter–chloramphenicol resistance gene (*cat*)–*P*_*gap*_ (from *P. limnophila*)–*gfpmut2*–downstream homology arm of *hpnD* (ca. 1500 bp). The transformation with the plasmid linearized outside of the insert sequence yielded a single white *S. maiorica* colony that showed the Δ*hpnD* phenotype (lack of pigmentation, genotype confirmed by check PCR) and a fluorescence signal in the cytoplasm (Fig. 3B). The protocol is hence suitable for the stable introduction of foreign DNA into more challenging strains. By choosing different homology regions, foreign DNA can also be inserted into intergenic regions and does not necessarily need to be combined with the inactivation of a protein-coding gene.

### Variation of the gene expression strength using alternative promoters in *P. limnophila*

**Fig. 4:**
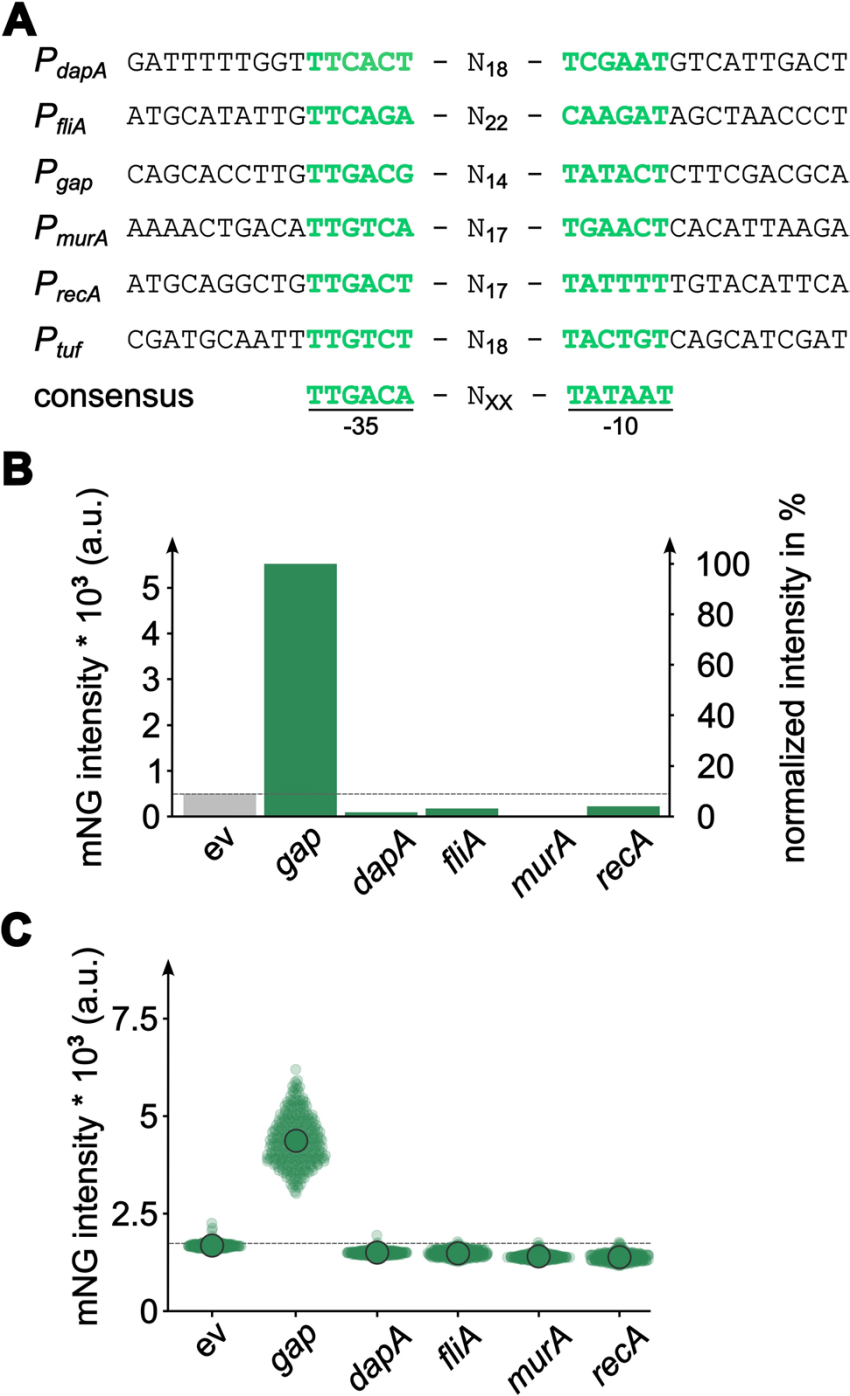
Testing of native promoters for alternation of the reporter gene expression levels in *P. limnophila*. **A** Predicted promoter sequences and comparison against the consensus sequence of σ^70^-dependent promoters. The predicted −35 and −10 regions of the promoters are shown in green. **B** Fluorescence intensity measurement on population level from cells harboring one of the selected promoter-reporter constructs. **C** Single-cell fluorescence intensity analysis of *P. limnophila* cells expressing the mNeonGreen reporter gene under the control of the selected promoters. Each circle represents one measured cell. ev, empty vector control; mNG, monomeric NeonGreen

After having established the protocol for the pTH13mod-based introduction of foreign DNA into the chromosome of *P. limnophila* and subsequent expression of fluorescent reporters, the next step included the testing of different native promoters aiming at different transcription rates of tested genes of interest. This becomes important for lowering the expression level of genes that code for proteins that show toxic effects to the host or interfere with essential cell biological processes, e.g., cell division. The glyceraldehyde-3-phosphate dehydrogenase promoter *P*_*gap*_ (also referred to as *gapdh* promoter) is frequently used in various genetically engineered bacteria because of its strong and constitutive expression during the exponential growth phase (Meyers et al. 2019; Olson et al. 2015; Pátek et al. 2003). Five different native *P. limnophila* promoters with assumingly lower natural transcription rates were chosen for the subsequent experiments in combination with mNeonGreen as reporter, namely *P*_*dapA*_ (4-hydroxytetrahydrodipicolinate synthase gene *dapA*, lysine biosynthesis) *P*_*murA*_ (UDP-*N*-acetylglucosamine 1-carboxyvinyltransferase gene *murA*, peptidoglycan biosynthesis), *P*_*recA*_* (recA* gene, involved in homologous recombination, DNA repair and competence), *P*_*fliA*_ (RNA polymerase sigma factor gene controlling the expression of flagella-related genes), and *P*_*tuf*_ (gene encoding the elongation factor Tu, protein biosynthesis) (Fig. 4A). The assumed expression mode of the genes is constitutive (*dapA*, *murA*, *tuf*), cell-cycle regulated (*fliA*, since the flagellum is only formed once the daughter cell is released from the mother cell), or stress-regulated (r*ecA*). To estimate the strength of the chosen promoters, the −35 and −10 regions predicted by bprom (Softberry Inc.) were compared against the consensus sequences of σ^70^-dependent promoters (Fig. 4A). Based on this analysis, *P*_*gap*_ showed the highest similarity to the consensus sequence (two mismatches) while *P*_*fliA*_, *P*_*murA*_, and *P*_*tuf*_ showed the lowest similarity (five mismatches each).

Correct clones could be obtained for all chosen promoters except *P*_*tuf*_, which did not give colonies of recombinant *P. limnophila* cells. To test the strength of the promoters in the obtained clones, fluorescence intensity was measured on a population level using a plate reader. As expected, the *P*_*gap*_ construct yielded the strongest fluorescence on population level; however, the fluorescence intensity of the other four promoters fell in the range of the autofluorescence of the empty vector control strain (Fig. 4B). The promoter strength could differ depending on the life cycle stage or stress level experienced by individual cells. Therefore, the fluorescence intensity was also checked on single-cell level employing wide-field fluorescence microscopy. The single-cell data showed a similar distribution of fluorescence levels, thereby confirming the results of the plate reader experiment (Fig. 4C).

### Commonly used inducible expression system do not function in *P. limnophila*

Inducible expression systems have decisive advantages over constitutive promoters in terms of flexibility. The time point of induction and the expression rate can be controlled by the externally supplemented inducer. The proper activity of an inducible expression system typically requires the synthesis of a functional transcriptional regulator protein, the efficient uptake of the inducing metabolite, and the recognition of the regulated promoter by the host’s transcription initiation complex. Plasmids harboring the following regulator/regulated promoter pairs were introduced into the genome of *P. limnophila* in combination with the msfTq2ox- and/or the mCherry-encoding gene: LacI/P_*tac*_ (inducer: isopropyl β-d−1-thiogalactopyranoside, IPTG) (Stansen et al. 2005), NagR/P_*nagAa*_ (inducer: salicylic acid) (Kruse et al. 2024), XylS/P_m_ (inducer: salicylic acid) (Hogenkamp et al. 2022), and TetR/P_*tetA*_ (inducer: anhydrotetracycline) (Skerra 1994). Although all plasmid-based systems were confirmed to be functional in *E. coli*, none of the systems worked in *P. limnophila*. Either no recombinant clones could be obtained after several rounds of transformation (both salicylate-inducible systems) or no expression of the reporter genes was detected even in presence of the maximal concentration of the inducers (LacI/P_*tac*_ and TetR/P_*tetA*_ systems) (data not shown).

### A native sugar-dependent regulator/promoter pair was turned into an inducible gene expression system

**Fig. 5:**
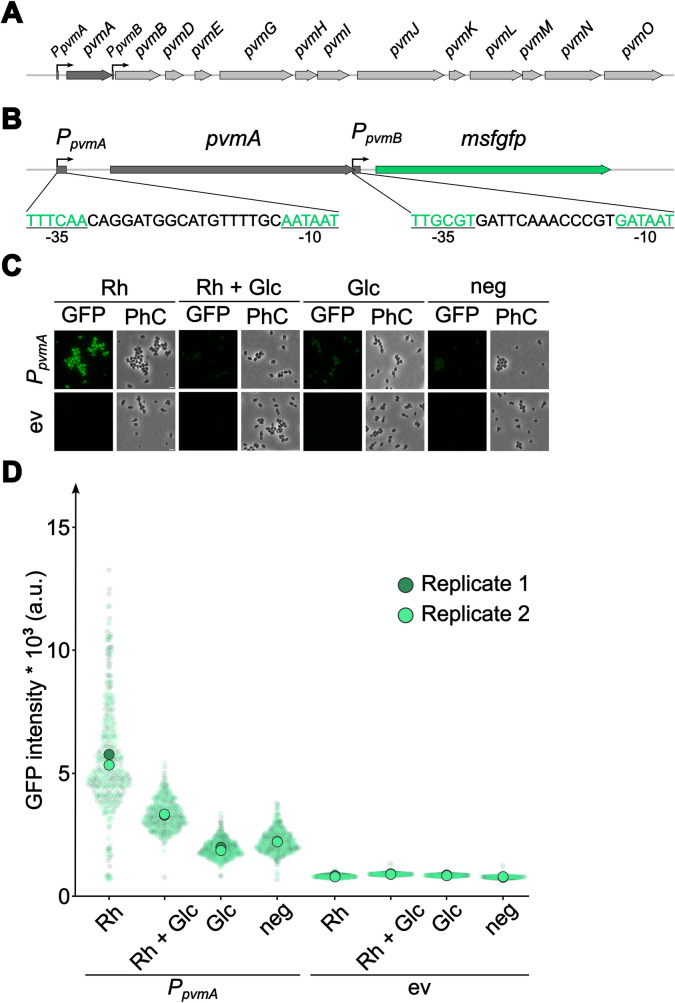
A native rhamnose-dependent promoter/regulator pair from *P. limnophila* displays differential expression of a fluorescent reporter when induced with rhamnose. **A** Genetic organization of the rhamnose catabolic cluster in *P. limnophila* including predicted promoters. **B** Organization of the constructed rhamnose promoter-reporter gene construct. The nucleotide sequence of the predicted −35 and −10 regions of the promoters are depicted in green. **C** Epi-fluorescence microscopy images showing altered expression of the reporter gene *msfgfp* upon induction of the promoter/regulator pair with rhamnose (Rh), glucose (Glc), a combination of rhamnose and glucose or without glucose or rhamnose. **D** Single-cell fluorescence intensity analysis of *P. limnophila* depicting the differential expression of *msfgfp* upon induction with rhamnose, glucose, the combination of rhamnose and glucose or without glucose or rhamnose. ev, empty vector control

**Fig. 6:**
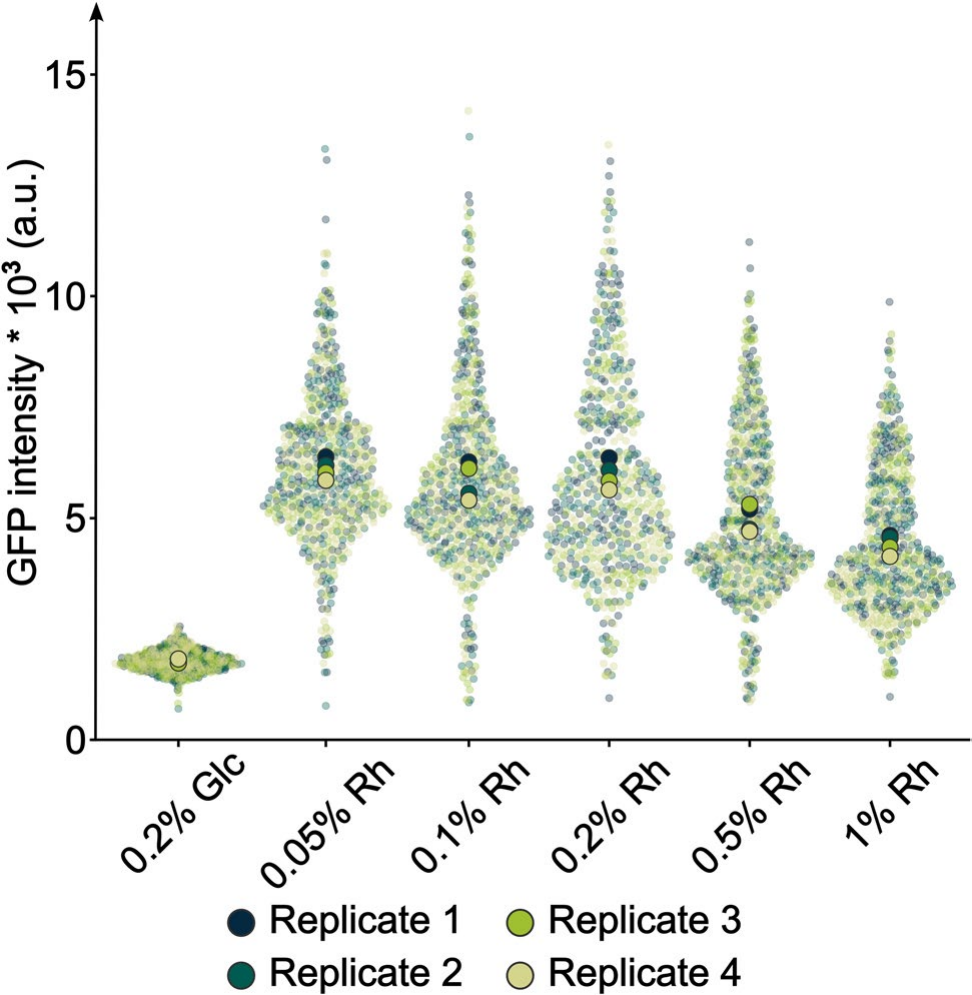
The expression level of the native rhamnose-dependent promoter/regulator pair depends on the rhamnose concentration. Fluorescence intensity single-cell analysis of *P. limnophila* cells when incubated with varying concentrations of rhamnose (in w/v). In total, four replicates with 150 cells each were analyzed. Glc, glucose; Rh, rhamnose

Since none of the commonly used inducible expression systems worked in *P. limnophila*, characterized regulatory circuits in this bacterium were evaluated as potential inducible gene expression systems. *P. limnophila* can use rhamnose and fucose as carbon and energy sources. The degradation of these monosaccharides has been shown to take place in a bacterial microcompartment (BMC) due to the toxicity of one of the pathway intermediates (Erbilgin et al. 2014). The genes constituting the rhamnose/fucose catabolic pathway are encoded along with BMC structural protein-encoding genes in the *pvmABDEGHIJKLMNO* cluster (Fig. 5A). The first gene of the cluster codes for the putative DeoR-family transcriptional regulator PvmA (Plim_1758, UniProt entry D5SXM1) that likely responds to rhamnose or one of the early pathway intermediates to induce the expression of the cluster. To turn the natural cluster into an inducible expression system, the predicted promoter regions and the open reading frame of *pvmA* were amplified and cloned into pTH13mod in a way that the start codon of the *msfgfp* gene is at the same position as that of *pvmB* in the original cluster (*pvmB* and subsequent genes were omitted) (Fig. 5B). The recombinant *P. limnophila* strain, now harboring P_*pvmA*_-*pvmA-P*_*pvmB*_*-msfgfp* in addition to the unmodified natural cluster, was cultivated in the presence of different concentrations and combinations of rhamnose and glucose as well as in the absence of an additional carbon source (Fig. 5B, C). Initial concentrations of 0.2% (w/v) rhamnose in the medium induced the expression of *msfgfp* and led to a strong fluorescence signal per cell that was homogeneously distributed throughout the cytoplasm (Fig. 5C, D) indicating a strong expression of the fluorescent reporter gene. When rhamnose was provided in addition to glucose, a lower expression level than for rhamnose alone was obtained; however, the expression was slightly higher than that in a culture without glucose or rhamnose. When only glucose was provided, the measured fluorescence was lower than that in a culture without glucose or rhamnose. This points towards the presence of additional catabolite repression mechanisms, suggesting a diauxic growth behavior of the strain. The presence of such a system appears physiologically reasonable since glucose degradation (glycolysis) does not require the additional expression of proteins forming a BMC and is therefore less costly. An empty vector control did not show any increase of the fluorescence signal over the autofluorescence level regardless of the amount of rhamnose or glucose present in the medium (Fig. 5D). To characterize the system in greater detail, the expression strength of the system was tested with different rhamnose concentrations ranging from 0.05 to 1% (Fig. 6). When 0.05%, 0.1%, or 0.2% rhamnose were supplied, the fluorescence intensity was similar (mean values of 6100 a.u., 5830 a. u., and 5970 a.u., respectively). Higher concentrations of rhamnose (0.5% and 1%) resulted in a decreased fluorescence intensity (mean values of 4990 a.u. and 4410 a.u., respectively), and the sole presence of 0.2% glucose showed a low fluorescence intensity (mean value of 1780 a.u.).

## Discussion

Planctomycetes are a group of bacteria that exhibit unique cellular and genomic features, making them intriguing but also challenging for genetic research. The current lack of robust genetic tools for planctomycetes can be attributed to several factors including their slow growth (generation times of at least 8 h), specialized growth requirements, limited knowledge of genetic mechanisms, inherent resistance to several classes of antibiotics, and a currently relatively small research community working with members of the phylum. Planctomycetes challenge many long-standing cell biological dogmas (Boedeker et al. 2017; Rivas-Marin et al. 2023) and their metabolism remains largely unexplored (Kallscheuer and Jogler 2021). To tackle open research questions, we here provide an improved molecular toolbox enabling the introduction of (multiple) genetic modifications and engineered (inducible) expression of (heterologous) genes.

The homologous recombination for deletion of genes by two simultaneous crossing-over events yielded more than 100 colonies in *P. limnophila* while the insertion-duplication mutagenesis approach for the insertion of entire plasmid cassettes worked one order of magnitude less efficient in our hands, typically yielding less than ten colonies per transformation reaction. The obtained differences are probably related to steric effects caused by the size of the plasmid compared to the shorter linearized fragment with up- and downstream homology regions. In addition, a competing excision reaction can occur after the single recombination event. This ultimately leads to the loss of the inserted plasmid sequence phenotypically visible in a lower number of obtained colonies. During the construction of deletion mutants, the number of obtained colonies typically decreased with the size of the inactivation target, *i.e.* the distance of the chosen up- and downstream homology arms. The crossing-over events appear to work best in cases in which the inactivation target is similar in length to the inserted resistance cassette. Larger inactivation targets might require secondary structure (hairpin) formation of the genomic DNA that could reduce the efficiency. For challenging strains, for which the less-efficient insertion-duplication mutagenesis fails, the gene deletion approach can also be exploited for the stable insertion of foreign DNA fragments (as shown for *gfpmut2* in *S. maiorica*).

The current need for the introduction of heterologous genes into the genome is based on the lack of a replicative plasmid for model strains. Plasmids are commonly found in members of the order *Isosphaerales* in the phylum (some strains harbor up to five plasmids) and even the type strain of *P. limnophila* harbors an extrachromosomal element (prophage). However, the exact elements required for plasmid replication and distribution to the daughter cell only start to be unveiled (Quiñonero-Coronel et al. 2024). Like for the tested expression systems, replication machineries of commonly used plasmids for *E. coli* or with broader host range do not work in *P. limnophila*.

Instead of the established gene expression systems, a native rhamnose-responsive genetic circuit was turned into an inducible expression system based on the rhamnose/fucose catabolic *pvm* operon in *P. limnophila*. The gene cluster in this species was studied in greater detail (Erbilgin et al. 2014), but is also present in several other members of the phylum including *S. maiorica*. The genetic organization suggests that *pvmA* (encoding a DeoR-family transcriptional regulator) might be part of the operon. Such an organization has, e.g., been observed in the DeoR-family regulator-encoding gene *rhaR* in the rhamnose catabolic operon in *Bacillus subtilis* (Hirooka et al. 2015). An indication for a similar genomic setup in *P. limnophila* is the short intergenic sequence of 46 bp between *pvmA* and *pvmB* that is much smaller compared to intergenic regions of many of the other genes in the predicted operon. However, the *pvmA*–*pvmB* intergenic region harbors a predicted promoter with the −35 and −10 region similar to the respective consensus sequences for σ^70^-dependent promoters. Thus, instead of the 13-gene operon, the cluster might be expressed as two transcripts, one monocistronic mRNA harboring *pvmA* and the other one harboring *pvmBDEGHIJKLMNO*. The exact mode of action of PvmA cannot be answered from the genetic organization alone; however, most of the characterized DeoR-family regulators including RhaR from *B. subtilis* act as repressors (Hirooka et al. 2015). After having cloned the rhamnose-inducible construct with *msfgfp* as reporter, it turned out that the gene was strongly expressed in *E. coli* without the addition of rhamnose. This might reinforce PvmA being a transcriptional repressor with insufficient expression of its encoding gene in *E. coli*. In the case of a transcriptional activator, no strong expression in *E. coli* would have been expected in the absence of rhamnose or with a sufficient or insufficient expression of *pvmA*. Although functional in *P. limnophila*, the strong expression of the target gene in *E. coli* restricts the system to genes that are non-toxic for the used *E. coli* cloning strain.

With the tested set of seven fluorescent reporter proteins active in *P. limnophila* (some efficiently maturing in the periplasm), additional planctomycete-derived genetic circuits should first be characterized and can then be tested as inducible gene expression systems. In parallel, efforts to construct *E. coli*–*P. limnophila* shuttle vectors need to be intensified to facilitate the future investigation of cell biological principles in these non-model bacteria.

## Supplementary Information

Below is the link to the electronic supplementary material.

Supplementary file1 (PDF 838 KB)

Supplementary file2 (XLSX 19.8 KB)

## Data Availability

Data and/or bacterial strains generated in this study are available upon reasonable request from the corresponding author. Nucleotide sequences of the used genes and promoters are provided in the Supporting Information.
