# Supplementary material for: Novel tools for genomic modification and heterologous gene expression in the phylum Planctomycetota
Source: Appl Microbiol Biotechnol. 2025 Mar 31;109:79. doi: 10.1007/s00253-025-13462-w (PMC11958385; doi:10.1007/s00253-025-13462-w)
Supplement: Supplementary file 1 [file 253_2025_13462_MOESM1_ESM.pdf]

Supporting Information

Novel tools for genomic modification and heterologous gene expression in the phylum *Planctomycetota*

Tom Haufschild[1], Jonathan Hammer[1], Nico Rabold[1], Veronika Plut[1], Christian Jogler[1,2,*] and Nicolai Kallscheuer[1,*]

1 - Department of Microbial Interactions, Institute for Microbiology, Friedrich Schiller University, 07743 Jena, Germany
2 - Cluster of Excellence Balance of the Microverse, Friedrich Schiller University, Jena, Germany

*Corresponding author:
Christian Jogler, e-mail: christian.jogler@uni-jena.de

Nicolai Kallscheuer, e-mail: nicolai.kallscheuer@uni-jena.de

Running Title: Novel genetic tools for planctomycetes

Keywords:

planctomycetes, *Planctopirus limnophila*, fluorescence proteins, inducible gene expression, gene inactivation, cell biology

Supporting Tables

The following supporting tables are provided as a separate Excel file:

Table S1. Strains and plasmids used in the study.

Table S2. Oligonucleotides used in the study.

Supporting Figures

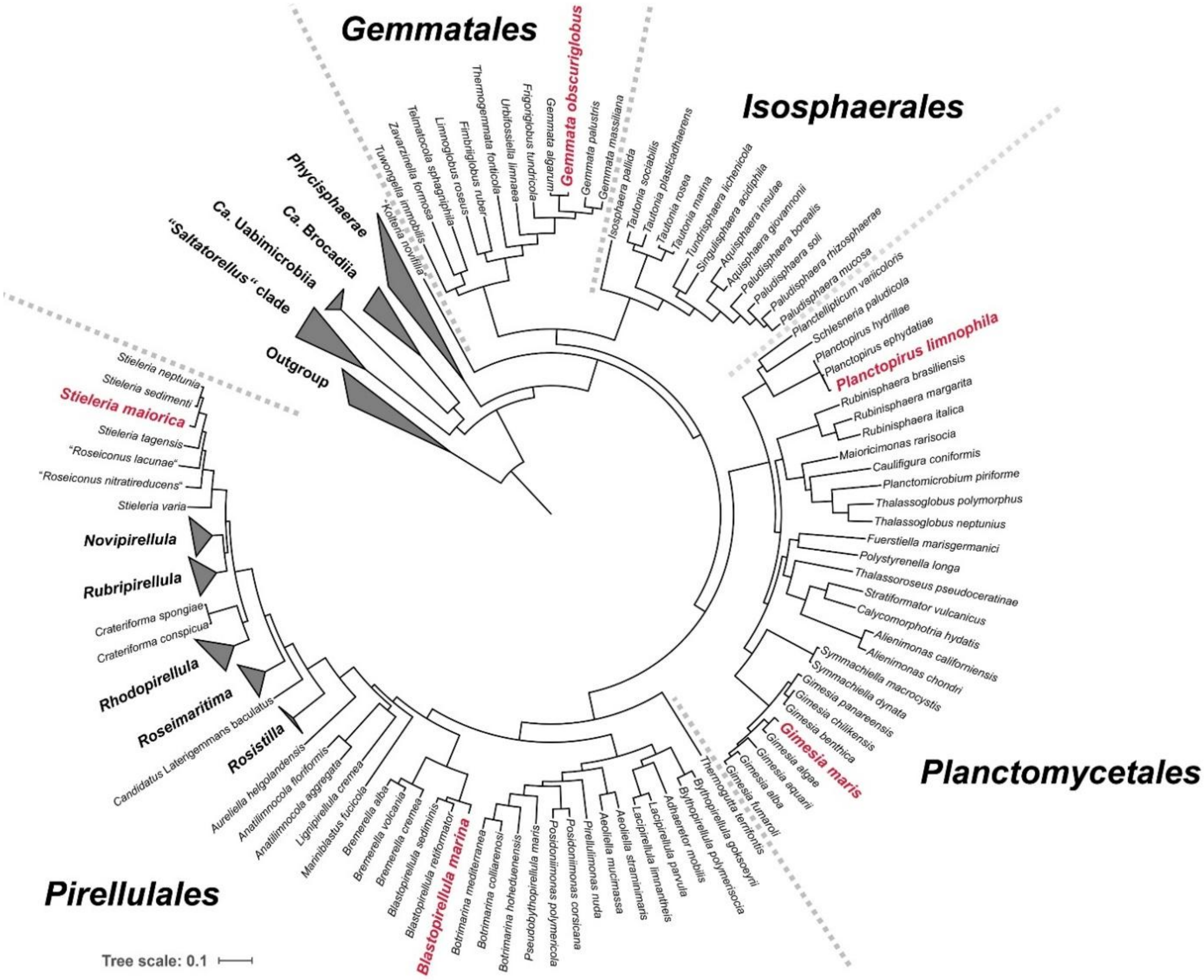

Figure S1. Phylogenetic tree highlighting the hitherto genetically modified planctomycetotal species. Multi-locus sequence analysis-based maximum likelihood phylogenetic tree based on all current type strains in the phylum *Planctomycetota*. Dashed lines delineate different orders. For simplicity, only the species names are shown on the branches while the names of the respective type strains were omitted. Species that have been genetically modified in this and previous studies are highlighted in red. The outgroup consists of three genomes of strains outside of the phylum *Planctomycetota*, but part of the *Planctomycetota-Verrucomicrobiota-Chlamydiota* (PVC) superphylum.

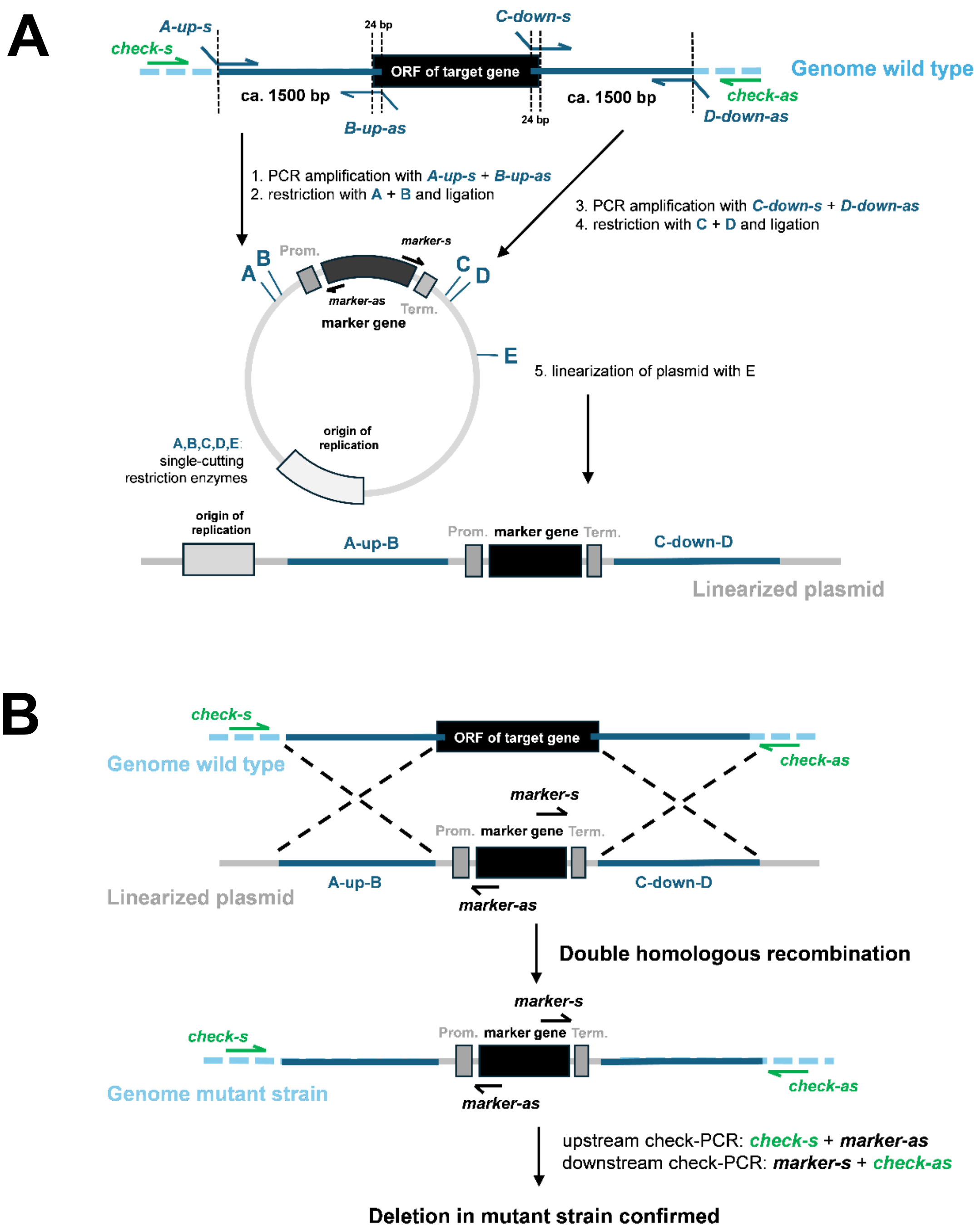

Figure S2. Gene deletion strategy via double homologous recombination. A) amplification and cloning steps: ca. 1500 bp flanking regions (including the first and last 24 bp of the open reading frame of the targeted gene) were amplified and cloned into the multiple cloning sites of a plasmid harboring the desired resistance marker gene that is later inserted into the chromosome in the deletion mutant. B) enforced double homologous recombination after transformation of the planctomycetotal strain.

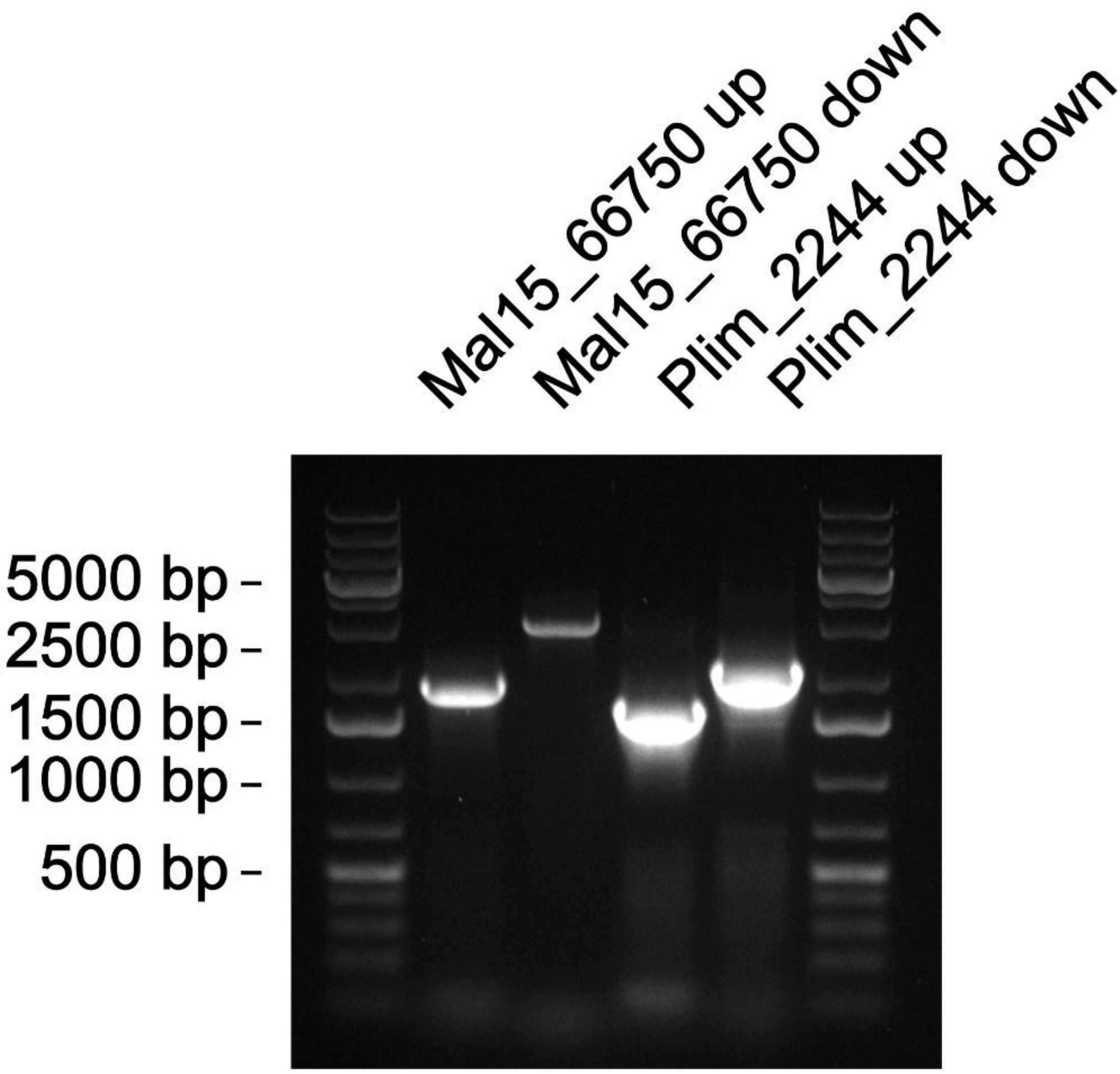

Figure S3. PCR-based verification of the *hpnD* deletion in *S. maiorica* and *P. limnophila*. PCR products of up- and downstream flanks of the *hpnD* deletion mutant strains with primers as shown in Fig. S2B confirmed the successful exchange of the native gene with the resistance cassette. Depending on the primers used, expected product sizes ranged between 1.7 and 3.2 kilobases.

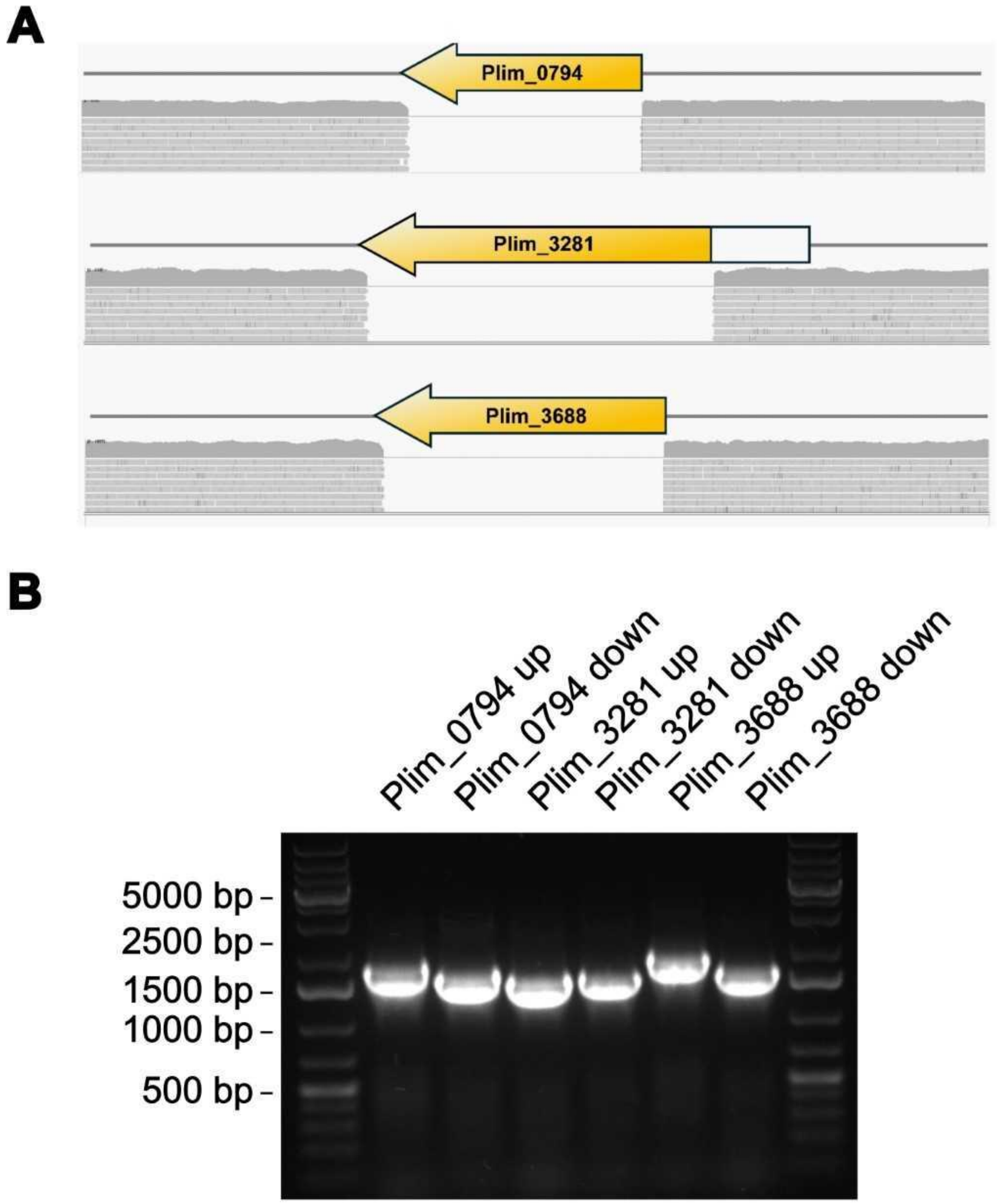

Figure S4. Verification of the *pilQ* deletions in *P. limnophila*. A) Obtained shorts reads of Illumina-sequenced genomic DNA of the *pilQ* single gene deletion mutants were mapped against the reference sequence (wild-type strain). The absence of reads in the coding region of the putative *pilQ* genes Plim_0794, Plim_3281 and Plim_3688 confirmed the expected single gene deletions. The non-filled white box indicates an alternative start codon in the automated RefSeq vs. GenBank-annotated genome of *P. limnophila*. B) PCR-based verification of up- and downstream flanks of the three genomic loci modified in the triple deletion mutant. Depending on the primers used (Fig. S2B), expected product sizes ranged between 1.7 and 1.9 kilobases.

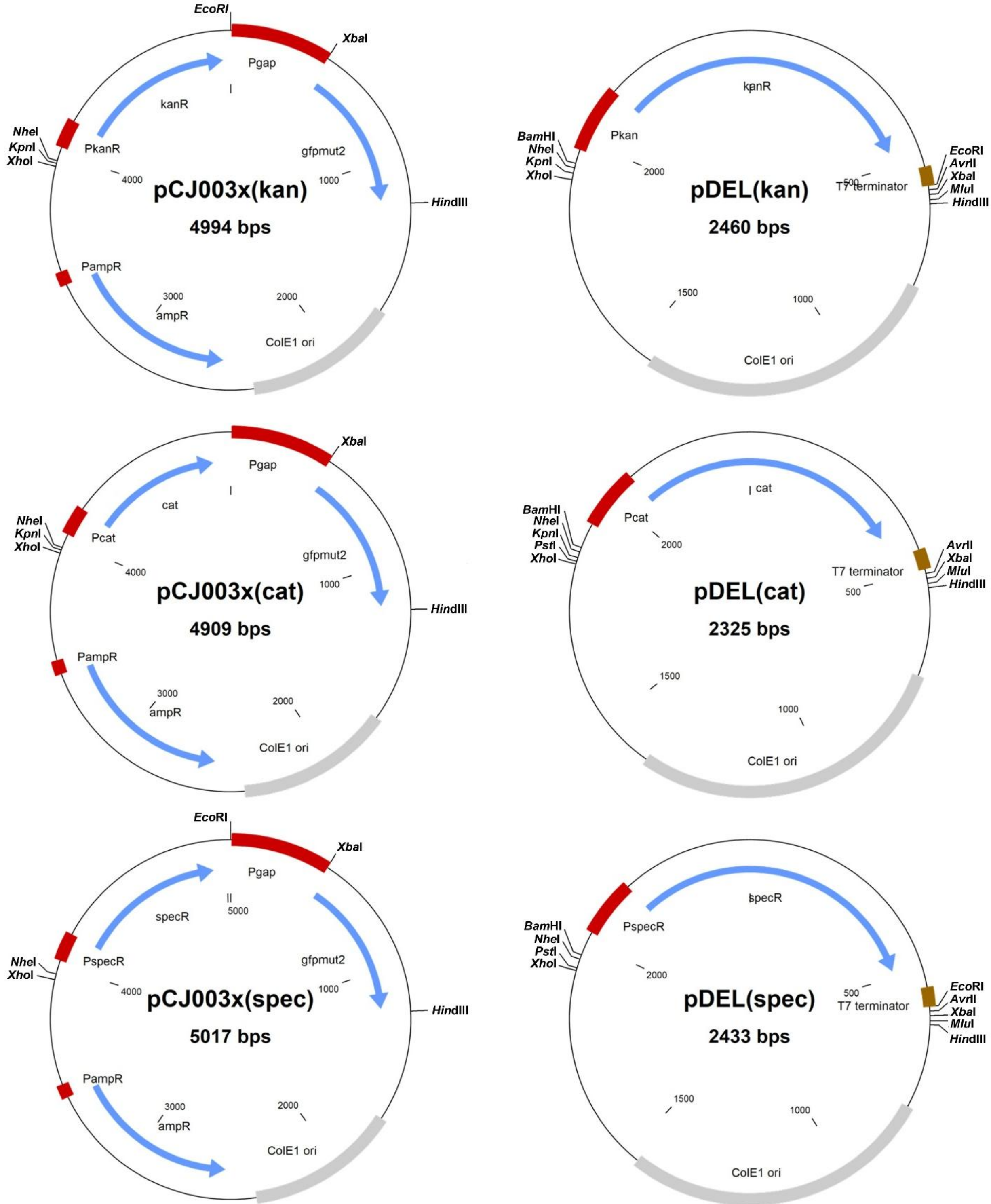

Figure S5. Plasmid maps of the pCJ003x and pDEL plasmids with different resistance marker genes. Unique sites for restriction enzymes available for cloning are shown. In the pCJ003x plasmids the P_{gap}-*gfpmut2* region was excised during cloning of the downstream homology region. kanR: kanamycin resistance gene, cat: chloramphenicol acetyltransferase (chloramphenicol resistance gene), specR: spectinomycin resistance gene, ampR: ampicillin resistance gene.

Nucleotide sequences used in the study

a) Gene sequences

```
>GFPmut2_gene
ATGAGTAAAGGAGAAGAACTTTTCACTGGAGTTGTCCCAATTCTTGTTGAATTAGATGGT
GATGTTAATGGGCACAAATTTTCTGTCAGTGGAGAGGGTGAAGGTGATGCAACATACGGA
AAACTTACCCTTAAATTTATTTGCACTACTGGAAAACTACCTGTTCCATGGCCAACACTT
GTCACTACTTTCGCGTATGGTCTTCAATGCTTTGCGAGATACCCAGATCATATGAAACAG
CATGACTTTTTCAAGAGTGCCATGCCCGAAGGTTATGTACAGGAAAGAACTATATTTTTC
AAAGATGACGGGAACTACAAGACACGTGCTGAAGTCAAGTTTGAAGGTGATACCCTTGTT
AATAGAATCGAGTTAAAAGGTATTGATTTTAAAGAAGATGGAAACATTCTTGGACACAAA
TTGGAATACAACTATAACTCACACAATGTATACATCATGGCAGACAAACAAAAGAATGGA
ATCAAAGTTAACTTCAAAATTAGACACAACATTGAAGATGGAAGCGTTCAACTAGCAGAC
CATTATCAACAAAATACTCCAATTGGCGATGGCCCTGTCCTTTTACCAGACAACCATTAC
CTGTCCACACAATCTGCCCTTTCGAAAGATCCCAACGAAAAGAGAGACCACATGGTCCTT
CTTGAGTTTGTAACAGCTGCTGGGATTACACATGGCATGGATGAACTATACAAATAA
```

```
>msfTq2ox_gene
ATGGTGAGCAAGGGCGAGGAGCTGTTCACCGGGGTGGTGCCCATCCTGGTCGAGCTGGAC
GGCGACGTAAACGGCCACAAGTTCAGCGTGCGCGGCGAGGGCGAGGGCGATGCCACCAAT
GGCAAGCTGACCCTGAAGTTCATCTGCACCACCGGCAAGCTGCCCGTGCCCTGGCCCACC
CTCGTGACCACCCTGTCCTGGGGTGTACAGGTCTTCGCCCGCTACCCCGACCACATGAAG
CAGCACGACTTCTTCAAGTCCGCCATGCCCGAAGGCTACGTCCAGGAGCGCACCATCAGC
TTCAAGGACGACGGCACATACAAGACCCGCGCCGAGGTGAAGTTCGAGGGCGACACCCTG
GTGAACCGCATCGAGCTGAAGGGCATCGACTTCAAGGAGGACGGCAACATCCTGGGGCAC
AAGCTGGAGTACAACTACTTTAGCGACAACGTCTATATCACCGCCGACAAGCAGAAGAAC
GGCATCAAGGCCAACTTCAAGATCCGCCACAACGTGGAGGACGGCGGCGTGCAGCTCGCC
GACCACTACCAGCAGAACACCCCCATCGGCGACGGCCCCGTGCTGCTGCCCGACAACCAC
TACCTGAGCACCCAGTCCAAGCTGAGCAAAGACCCCAACGAGAAGCGCGATCACATGGTC
CTGCTGGAGTTCGTGACCGCCGCCGGGATCACTCTCGGCATGGACGAGCTGTACAAGTAA
```

```
>msfGFP_gene
ATGAGCAAAGGCGAAGAACTGTTTACCGGCGTGGTGCCGATTCTGGTGGAACTGGATGGC
GATGTGAACGGCCATAAATTTAGCGTGCGCGGCGAAGGCGAAGGCGATGCGACCAACGGC
AAACTGACCCTGAAATTTATTTGCACCACCGGCAAACTGCCGGTGCCGTGGCCGACCCTG
GTGACCACCCTGACCTATGGCGTGCAGTGCTTTAGCCGCTATCCGGATCATATGAAACGC
CATGATTTTTTTAAAAGCGCGATGCCGGAAGGCTATGTGCAGGAACGCACCATTAGCTTT
AAAGATGATGGCACCTATAAAACCCGCGCGGAAGTGAAATTTGAAGGCGATACCCTGGTG
AACCGCATTGAACTGAAAGGCATTGATTTTAAAGAAGATGGCAACATTCTGGGCCATAAA
CTGGAATATAACTTTAACAGCCATAACGTGTATATTACCGCGGATAAACAGAAAAACGGC
ATTAAAGCGAATTTTAAAACCCGCCATAACGTGGAAGATGGCAGCGTGCAGCTGGCGGAT
CATTATCAGCAGAACACCCCGATTGGCGATGGCCCGGTGCTGCTGCCGGATAACCATTAT
CTGAGCACCCAGAGCAAGCTGAGCAAAGATCCGAACGAAAAACGCGATCATATGGTGCTG
CTGGAATTTGTGACCGCGGCGGGCATTACCCACGGCATGGATGAACTGTATAAATAA
```

>mNeonGreen_gene
ATGGTGAGCAAGGGCGAGGAGGATAACATGGCCTCTCTCCCAGCGACACATGAGTTACAC
ATCTTTGGCTCCATCAACGGTGTGGACTTTGACATGGTGGGTCAGGGCACCGGCAATCCA
AATGATGGTTATGAGGAGTTAAACCTGAAGTCCACCAAGGGTGACCTCCAGTTCTCCCCC
TGGATTCTGGTCCCTCATATCGGGTATGGCTTCCATCAGTACCTGCCCTACCCTGACGGG
ATGTCGCCTTTCCAGGCCGCCATGGTAGATGGCTCCGGCTACCAAGTCCATCGCACAATG
CAGTTTGAAGATGGTGCCTCCCTTACTGTTAACTACCGCTACACCTACGAGGGAAGCCAC
ATCAAAGGAGAGGCCCAGGTGAAGGGGACTGGTTTCCCTGCTGACGGTCCTGTGATGACC
AACTCGCTGACCGCTGCGGACTGGTGCAGGTCGAAGAAGACTTACCCCAACGACAAAACC
ATCATCAGTACCTTTAAGTGGAGTTACACCACTGGAAATGGCAAGCGCTACCGGAGCACT
GCGCGGACCACCTACACCTTTGCCAAGCCAATGGCGGCTAACTATCTGAAGAACCAGCCG
ATGTACGTGTTCCGTAAGACGGAGCTCAAGCACTCCAAGACCGAGCTCAACTTCAAGGAG
TGGCAAAAGGCCTTTACCGATGTGATGGGCATGGACGAGCTGTACAAGTAA

>mVenus_gene
ATGGTGAGCAAGGGCGAGGAGCTGTTCACCGGGGTGGTGCCCATCCTGGTCGAGCTGGAC
GGCGACGTAAACGGCCACAAGTTCAGCGTGTCCGGCGAGGGCGAGGGCGATGCCACCTAC
GGCAAGCTGACCCTGAAGCTGATCTGCACCACCGGCAAGCTGCCCGTGCCCTGGCCCACC
CTCGTGACCACCCTGGGCTACGGCCTGCAGTGCTTCGCCCGCTACCCCGACCACATGAAG
CAGCACGACTTCTTCAAGTCCGCCATGCCCGAAGGCTACGTCCAGGAGCGCACCATCTTC
TTCAAGGACGACGGCAACTACAAGACCCGCGCCGAGGTGAAGTTCGAGGGCGACACCCTG
GTGAACCGCATCGAGCTGAAGGGCATCGACTTCAAGGAGGACGGCAACATCCTGGGGCAC
AAGCTGGAGTACAACTACAACAGCCACAACGTCTATATCACCGCCGACAAGCAGAAGAAC
GGCATCAAGGCCAACTTCAAGATCCGCCACAACATCGAGGACGGCGGCGTGCAGCTCGCC
GACCACTACCAGCAGAACACCCCCATCGGCGACGGCCCCGTGCTGCTGCCCGACAACCAC
TACCTGAGCTACCAGTCCAAGCTGAGCAAAGACCCCAACGAGAAGCGCGATCACATGGTC
CTGCTGGAGTTCGTGACCGCCGCCGGGATCACTCTCGGCATGGACGAGCTGTACAAGTAA

>mCherry_gene
ATGGTGAGCAAGGGCGAGGAGGATAACATGGCCATCATCAAGGAGTTCATGCGCTTCAAG
GTGCACATGGAGGGCTCCGTGAACGGCCACGAGTTCGAGATCGAGGGCGAGGGCGAGGGC
CGCCCCTACGAGGGCACCCAGACCGCCAAGCTGAAGGTGACCAAGGGTGGCCCCCTGCCC
TTCGCCTGGGACATCCTGTCCCCTCAGTTCATGTACGGCTCCAAGGCCTACGTGAAGCAC
CCCGCCGACATCCCCGACTACTTGAAGCTGTCCTTCCCCGAGGGCTTCAAGTGGGAGCGC
GTGATGAACTTCGAGGACGGCGGCGTGGTGACCGTGACCCAGGACTCCTCCCTGCAGGAC
GGCGAGTTCATCTACAAGGTGAAGCTGCGCGGCACCAACTTCCCCTCCGACGGCCCCGTA
ATGCAGAAGAAGACCATGGGCTGGGAGGCCTCCTCCGAGCGGATGTACCCCGAGGACGGC
GCCCTGAAGGGCGAGATCAAGCAGAGGCTGAAGCTGAAGGACGGCGGCCACTACGACGCT
GAGGTCAAGACCACCTACAAGGCCAAGAAGCCCGTGCAGCTGCCCGGCGCCTACAACGTC
AACATCAAGTTGGACATCACCTCCCACAACGAGGACTACACCATCGTGGAACAGTACGAA
CGCGCCGAGGGCCGCCACTCCACCGGCGGCATGGACGAGCTGTACAAGTAA

```
>HaloTag_gene
ATGTCCGAAATCGGTACTGGCTTTCCATTCGACCCCCATTATGTGGAAGTCCTGGGCGAG
CGCATGCACTACGTCGATGTTGGTCCGCGCGATGGCACCCCTGTGCTGTTCCTGCACGGT
AACCCGACCTCCTCCTACGTGTGGCGCAACATCATCCCGCATGTTGCACCGACCCATCGC
TGCATTGCTCCAGACCTGATCGGTATGGGCAAATCCGACAAACCAGACCTGGGTTATTTC
TTCGACGACCACGTCCGCTTCATGGATGCCTTCATCGAAGCCCTGGGTCTGGAAGAGGTC
GTCCTGGTCATTCACGACTGGGGCTCCGCTCTGGGTTTCCACTGGGCCAAGCGCAATCCA
GAGCGCGTCAAAGGTATTGCATTTATGGAGTTCATCCGCCCTATCCCGACCTGGGACGAA
TGGCCAGAATTTGCCCGCGAGACCTTCCAGGCCTTCCGCACCACCGACGTCGGCCGCAAG
CTGATCATCGATCAGAACGTTTTTATCGAGGGTACGCTGCCGATGGGTGTCGTCCGCCCG
CTGACTGAAGTCGAGATGGACCATTACCGCGAGCCGTTCCTGAATCCTGTTGACCGCGAG
CCACTGTGGCGCTTCCCAAACGAGCTGCCAATCGCCGGTGAGCCAGCGAACATCGTCGCG
CTGGTCGAAGAATACATGGACTGGCTGCACCAGTCCCCTGTCCCGAAGCTGCTGTTCTGG
GGCACCCCAGGCGTTCTGATCCCACCGGCCGAAGCCGCTCGCCTGGCCAAAAGCCTGCCT
AACTGCAAGGCTGTGGACATCGGCCCGGGTCTGAATCTGCTGCAAGAAGACAACCCGGAC
CTGATCGGCAGCGAGATCGCGCGCTGGCTGTCTACTCTGGAGATTTCCGGTTAA

>pvmA_gene_Planctopirus_limnophila
ATGCTTTTGGATCAACGCCGTCGACATGTGCTCGAACTCATTGAAGAAAAAGGCTTTATA
TCGCTTCATGAGCTTGCGACCAAAACCGGGGTCAGCGAGTCAACACTGAGGCGGGATTTG
GAATATCTTGACGGAATTCGGCAGGTTCGCCGCACACGAGGCGGGGCTGCCTATGTCGGT
GAATCGGTTGTTTCTCTGGAAGAGCGAAGTGTGACCTCTCTGGTCGAGAAGCAGAGAATT
GCTCGTTCGATCTCGGAAACGATAGGAAGTGGTGAAACAGTCCTGCTCGATGGGGGAACG
ACAACGCTTGAGGTGGCTCGTGCCTTGATTGGCAAAGAACTGCAAGTTGTGACCAATTCC
CTGGCGATTGCCAATTTGCTGGTGAACAGCCCGGGTGTGGAATTGATTTTTTTAGGAGGG
TATCTGCATCCCAAAACAGGTGTGACTTTAGGCCCGCTCCTGAATTTGGCGTTATCGCAA
TTGCAGGTCCCTCGCATGGTTTTCAGTGTCGGTGGCGTGACGGAGAAAGGCTTGTTCAAC
AGCAACACCCTCCTCGTTGAGGCCGAGCGGCGGATGATTGATGCCGCTGAACGAGTGGTG
CTGGCGGTTGATAGTCGGAAGTTTGGAAAAGCAGCCCTCTCCCCACTTTGTCCACTCGAC
CGTGTTCATGAAATTGTGACAGACGAAGGGATTCCCGAAGATTGGCGAAAGCGGATTGAA
GACTTGGGAATTGAACTGCGAATTGCGTGA

>nagR_gene_Comamonas_testosteroni
ATGGATCTGCGCGACATCGACTTGAATCTGCTGGTGGTCTTCAACCAGCTACTGCTCGAC
CGGAGCGTATCGACGGCCGGCGAAAAACTGGGGCTGACGCAGCCTGCCGTCAGCAATTCA
CTTAAACGGCTGCGTGCGGCGCTAAAGGACGATTTGTTCTTGCGCACCTCAAAAGGCATG
GAGCCGACACCGTATGCACTGCATCTTGCGGAGCCCGTGATCTATGCGCTCAACACGCTG
CAGACGGCACTGACGACCCGTGACTCTTTCGACCCATTTGCCAGCACGCGCACCTTCAAC
TTGGCAATGACCGACATCGGCGAGATGTACTTCATGCCCCCACTGATGGAAGCGCTTGCG
CAACGAGCTCCTCACATCCAGATCAGCACGCTGCGCCCGAATGCTGGCAATCTGAAGGAG
GATATGGAGTCCGGTGCGGTTGATCTCGCCTTGGGTCTTCTGCCAGAGCTACAGACCGGA
TTCTTCCAGCGGCGCCTCTTTCGCCACCGCTACGTATGCATGTTCCGCAAGGACCATCCA
AGCGCCAAATCCCCCATGAGCCTGAAACAGTTCAGTGAACTGGAGCATGTCGGCGTGGTC
GCACTCAACACCGGACACGGTGAGGTCGATGGCCTGCTCGAACGCGCAGGCATCAAAAGG
CGCATGCGGCTGGTGGTGCCGCATTTCATTGCGATCGGCCCCATTCTGCACAGCACCGAC
CTCATCGCGACCGTGCCGCAGCGTTTTGCCGTTCGCTGCGAAGTGCCTTTTGGTCTGACG
ACATCCCCGCACCCGGCCAAGCTGCCCGACATCGCCATCAACCTGTTTTGGCATGCCAAG
TACAACCGGGATCCGGGCAACATGTGGCTACGTCAGTTGTTCGTCGAGCTTTTCTCTGAA
GCATAA
```

```
>xylS_gene_Pseudomonas_putida
ATGGATTTTTGCTTATTGAACGAGAAAAGTCAGATCTTCGTCCACGCCGAGCCCTATGCA
GTCTCCGATTATGTTAACCAGTATGTCGGTACGCACTCTATTCGCCTGCCCAAGGGCGGG
CGCCCGGCAGGCACGCTGCACCACAGAATCTTCGGATGCCTCGACCTGTGTCGAATCAGC
TACGGCGGTAGCGTGAGGGTAATCTCGCCTGGATTAGAGACCTGTTATCATCTGCAAATA
ATACTCAAAGGCCATTGCCTGTGGCGTGGCCATGGCCAGGAGCACTATTTTGCGCCGGGC
GAACTATTGCTGCTCAATCCGGATGACCAAGCCGACCTGACCTATTCAGAAGATTGCGAG
AAATTTATCGTTAAATTGCCCTCAGTGGTCCTTGATCGGGCATGCAGTGACAACAATTGG
CACAAGCCGAGGGAGGGTATCCGTTTCGCCGCGCGACACAATCTCCAGCAACTCGATGGC
TTTATCAATCTACTCGGGTTAGTTTGTGACGAAGCGGAACATACAAAGTCGATGCCTCGG
GTCCAAGAGCACTATGCGGGGATCATCGCTTCCAAGCTGCTCGAAATGCTGGGCAGCAAT
GTCAGCCGTGAAATTTTCAGCAAAGGTAACCCGTCTTTCGAGCGAGTCGTTCAATTCATT
GAGGAGAATCTCAAACGGAATATCAGCCTTGAGCGGTTAGCGGAGCTGGCGATGATGAGT
CCACGCTCGCTCTACAATTTGTTCGAGAAGCATGCCGGCACCACGCCGAAGAACTACATC
CGCAACCGCAAGCTCGAAAGCATCCGCGCCTGCTTGAACGATCCCAGTGCCAATGTGCGT
AGTATAACTGAGATAGCCCTAGACTACGGCTTCTTACATTTGGGACGCTTCGCTGAAAAC
TATAGGAGCGCGTTCGGCGAGTTGCCTTCCGACACCCTGCGTCAATGCAAAAAGGAAGTG
GCTTGA

>lacI_gene
ATGGCGGAGCTGAATTACATTCCCAACCGCGTGGCACAACAACTGGCGGGCAAACAGTCG
TTGCTGATTGGCGTTGCCACCTCCAGTCTGGCCCTGCACGCGCCGTCGCAAATTGTCGCG
GCGATTAAATCTCGCGCCGATCAACTGGGTGCCAGCGTGGTGGTGTCGATGGTAGAACGA
AGCGGCGTCGAAGCCTGTAAAGCGGCGGTGCACAATCTTCTCGCGCAACGCGTCAGTGGG
CTGATCATTAACTATCCGCTGGATGACCAGGATGCCATTGCTGTGGAAGCTGCCTGCACT
AATGTTCCGGCGTTATTTCTTGATGTCTCTGACCAGACACCCATCAACAGTATTATTTTC
TCCCATGAAGACGGTACGCGACTGGGCGTGGAGCATCTGGTCGCATTGGGTCACCAGCAA
ATCGCGCTGTTAGCGGGCCCATTAAGTTCTGTCTCGGCGCGTCTGCGTCTGGCTGGCTGG
CATAAATATCTCACTCGCAATCAAATTCAGCCGATAGCGGAACGGGAAGGCGACTGGAGT
GCCATGTCCGGTTTTCAACAAACCATGCAAATGCTGAATGAGGGCATCGTTCCCACTGCG
ATGCTGGTTGCCAACGATCAGATGGCGCTGGGCGCAATGCGCGCCATTACCGAGTCCGGG
CTGCGCGTTGGTGCGGATATCTCGGTAGTGGGATACGACGATACCGAAGACAGCTCATGT
TATATCCCGCCGTTAACCACCATCAAACAGGATTTTCGCCTGCTGGGGCAAACCAGCGTG
GACCGCTTGCTGCAACTCTCTCAGGGCCAGGCGGTGAAGGGCAATCAGCTGTTGCCCGTC
TCACTGGTGAAAAGAAAAACCACCCTGGCGCCCAATACGCAAACCGCCTCTCCCCGCGCG
TCGGCCGCCATGCCGGCGATAATGGCCTGCTTCTCGCCGAAACGTTTGGTGGCGGGACCA
GTGACGAAGGCTTGA

>tetR_gene
ATGATGTCTCGTTTAGATAAAAGTAAAGTGATTAACAGCGCATTAGAGCTGCTTAATGAG
GTCGGAATCGAAGGTTTAACAACCCGTAAACTCGCCCAGAAGCTAGGTGTAGAGCAGCCT
ACATTGTATTGGCATGTAAAAAATAAGCGGGCTTTGCTCGACGCCTTAGCCATTGAGATG
TTAGATAGGCACCATACTCACTTTTGCCCTTTAGAAGGGGAAAGCTGGCAAGATTTTTTA
CGTAATAACGCTAAAAGTTTTAGATGTGCTTTACTAAGTCATCGCGATGGAGCAAAAGTA
CATTTAGGTACACGGCCTACAGAAAAACAGTATGAAACTCTCGAAAATCAATTAGCCTTT
TTATGCCAACAAGGTTTTTCACTAGAGAATGCATTATATGCACTCAGCGCAGTGGGGCAT
TTTACTTTAGGTTGCGTATTGGAAGATCAAGAGCATCAAGTCGCTAAAGAAGAAAGGGAA
ACACCTACTACTGATAGTATGCCGCCATTATTACGACAAGCTATCGAATTATTTGATCAC
CAAGGTGCAGAGCCAGCCTTCTTATTCGGCCTTGAATTGATCATATGCGGATTAGAAAAA
CAACTTAAATGTGAAAGTGGGTCTTAA
```

b) Promoter sequences

Predicted -35 and -10 regions are underlined.

```
>PdapA
CACTCGCAGAACAATTGTATCTCTCAGTGAAAGCCAAAGGTTTTGGCCGCAATGGGACTC
ACGCATTGATGCTGGCACTTGCAGAAATGTCCAATATCGATTGGCGCAACCGTACCAGCA
ATTCGTAAACACAAAGGGCCTGATGATCCCGGATAGGACTGGTCATTACGGGGTTTCTCT
CGGACAATTTCACTTTCAGGCCCGATCGTTACATCGAGATTTTTGGTTTCACTTACTACT
GATACCCGTCCTCGAATGTCATTGACTGCGAGTATCAGGCTTTCTTGAGGGTTGTTGAAA

>PfliA
TCGAAGGGAGTGGATTTATAGAGACTGTCAGAAAAGGTCTGATAGCGATGATGATCTTCT
TTCCGCTGGGCTGGTTCCTGGGAGAACTGGGTCGCCAGACAGCGGAGGATATGGCCAGAA
AAGATCTGACAAGAAAAGATCTGGCTGATTCATCGCCTGGACTGCCGACTGACAGTGTCA
CTGCCCGATAGTTAGTTATTTATCTGCGGCTCTATGCATATTGTTCAGAACCGCATTCGC
CACGATGCAAGCAAGATAGCTAACCCTCATTAGCTATCACGGATGTTCACGGAGGGTTGG

>Pgap
CCATCACCTGTTGAGGCGATTCGCTGCCGCATGTTCCCGTGGTCGCCATGATGATCACTC
CGTATTCATTCCAAGGACAATCAACGATCCATGAAGGCAGTGGCCACACCAATGGCTGAT
AACACGGCAGGATCGAACTTCTTTACACGATTACCCTGACAATATCGCGTGGAATGTTAT
CGACTTTCAATGCCATAATGTTTCCTTATGAGAAAATTCATCGAGTCGGTGAACTTGAAC
TTGCGCGGCCTGTTGAGGCTGTGTGGAATTGACGTCGAGATGGTGAGAAGCAGTTATCGC
ATTGGTGATGGTGGATGCGTTGTGTCAGCACCTTGTTGACGGCATTGCGCAGCCGTATAC
TCTTCGACGCATAAGAAAGATCGTCTGCGAACTGTCCGACTTCGTTGTTGAGACATGTCC
AGACTGTACTTCAGTTG

>PmurA
ATGTTTCCAATCGAGACCCGATGTACGATGATCGACTCGGGTTAAACGTCACCTGGTTTG
CCAGCTTGATCCCGCAGATTATCGAAGGAATACTTCCAGCAAATCCAAATCCAACGCCGA
CCAGCGATTCGGTTTGCTTCAATTAATCTCGAAGCTCAGAACTCAAGTCTCGATCAAAGG
TAACAGTCTTAAGAAAACTGACATTGTCAACAATTGTTTGTCATGTTGAACTCACATTAA
GATCATGTGGGGCAAGGTAAGTCATTGACTTACCAGATTGAAATCCAGAGGAGTCCAAGG

>PrecA
GGAATCTGCAGAAATCACCATGCTGCCGCAGAACACGGTCGAGCTTGATCTGGAGATGGG
GCGAGCCGTCCTGGCTTTAATTGATGCTCTCGAAGACAACGATGACATTCAGAATGTGAT
GTCGAATTTCAGCGTGTCGGAAGAAGTGTTGGCAGAGCTTTCGAAGTGATCAAACGAAAT
CCATTTCATGCAGGCTGTTGACTGTTCATTTGGTTTCTTGTATTTTTGTACATTCACGAT
GAATGTGGCTGCGTACGGATTGTGATTGACCGGAAAAGATTTCAAAAAGGATGACTCAGG

>Ptuf
CGTAGCTCAGTTGGTAGAGTGCGTCCTTGGTAAGGACGAGGTCGTGGGTTCGAATCCCAC
CGGTAGCTTTTGGTGGCGAGATTCTGGCAAGGTTGCCAGTCTTTACACGATGCAATTTTG
TCTGGATGTTGGCAGTTTGGTTACTGTCAGCATCGATTGAGTGTTTGAGTCCACGTATTT
GGCGTGGGTGCCGTCGTGGTGCCCATGGGATTGTCGTCCTCTCGTTCTGTCGTCCCAATT
TGATACACGGGCGGAAAGTTCCGCTGGTCGACAAACAGCAACAAAGAAGGTTTCGGAGAA
```

Underlined regions: PdapA TTCACT, TCGAAT; PfliA TTCAGA, CAAGAT; Pgap TTGACG, TATACT; PmurA TTGTCA, TGAACT; PrecA TTGACT, TATTTT; Ptuf TTGTCT, TACTGT.
